# Analysis of the implementation of nutritional care in the context of Primary Health Care

**DOI:** 10.11606/s1518-8787.2026060006929

**Published:** 2026-09-09

**Authors:** Patrícia Maria de Oliveira Machado, Josimari Telino de Lacerda, Rafaela Souza Petrolini, Mick Lennon Machado

**Affiliations:** I Universidade Federal de Santa Catarina Centro de Ciências da Saúde Departamento de Saúde Pública Florianópolis SC Brasil Universidade Federal de Santa Catarina. Centro de Ciências da Saúde. Departamento de Saúde Pública. Florianópolis, SC, Brasil

**Keywords:** Health Evaluation, Nutrition and Food Programs and Policies, Health Services Research

## Abstract

**OBJECTIVE:**

To evaluate the implementation of nutritional care within the Primary Health Care (PHC) system in Brazilian municipalities and to identify the association between the degree of implementation and aspects of the sociodemographic and political-organizational context.

**METHODS:**

This is an evaluative study with a quantitative approach that examined the responsibility of municipal managers in ensuring conditions and implementing interventions, based on the analysis of 17 indicators grouped into the dimensions of management and care. The municipalities were classified according to the degree of implementation of nutritional care in PHC by assessing the observed score relative to the maximum score, using the criteria “incipient,” “intermediate,” and “advanced.” The influence of the context on the degree of implementation was analyzed, with the independent variables grouped into two blocks, using multivariate logistic regression.

**RESULTS:**

A total of 4,064 Brazilian municipalities were evaluated, with an advanced level of implementation observed in 11.6% (n = 471). Management aspects received lower scores compared to care aspects. The contextual factors associated with a higher level of implementation of nutritional care were municipalities with a population of fewer than 25,000 inhabitants, located in the Southern Region, better social indicators, a lower proportion of children under five years of age, 100% coverage by the Family Health Strategy, and support from a dietitian in PHC.

**CONCLUSION:**

The implementation of nutritional care in Brazilian PHC was considered in its early stages during the period under investigation. Despite significant advances in food and nutrition surveillance, nutritional care exhibited major weaknesses in the management and coordination of care. We reiterate the need for continuous processes to evaluate and monitor interventions in Primary Health Care, improve the quality of care, and integrate nutritional care into the care model.

## INTRODUCTION

Nutritional care is defined as the set of food and nutrition (FN) services aimed at the promotion, prevention, diagnosis, and treatment of health conditions^1^. It is recognized as a key pillar of care and finds in Primary Health Care (PHC) a strategic setting for its implementation and consolidation^2,3^.

PHC is considered the preferred entry point to the Health Care Network (HCN) because it offers a high degree of reach and greater proximity to individuals and families in local communities^4^. In this context, comprehensive or integrated PHC differs from selective PHC, which focuses on limited packages of low-cost interventions for specific population groups and isolated health conditions^5^. Comprehensive or integrated PHC is based on the health needs of the population and the comprehensiveness of care, reflecting its potential to address the current epidemiological and nutritional situation^4^.

Despite the regulatory frameworks that integrate nutritional care into the Brazilian health system—such as the Política Nacional de Alimentação e Nutrição (PNAN – National Food and Nutrition Policy)^1^, which establishes the organization of nutritional care in the HCN, and the Política Nacional de Atenção Básica (National Primary Care Policy)^4^, which defines nutrition as a cross-cutting component of the Estratégia Saúde da Família (ESF – Family Health Strategy), the ideal model of comprehensive PHC still faces barriers. The numerous nutritional care programs and initiatives are characterized by the persistence of a programmatic, assistance-oriented approach.

Programmatic initiatives are characterized by the organization of services around specific health conditions and prescriptive technical standards, creating barriers to the implementation of a care model based on comprehensiveness. This occurs due to the tendency of these initiatives to fragment care and their limited ability to adapt to the different political-organizational, socioeconomic, and demographic contexts of the regions^6^.

The persistence of the programmatic approach is particularly critical in the context of Brazil’s nutritional transition, marked by the coexistence of malnutrition and the rapid rise in obesity and other chronic noncommunicable diseases. The absence of effectively implemented nutritional care within PHC results in reactive, disease-centered care that is unable to address contemporary health problems. Thus, it is essential to develop evaluation models for the implementation of nutritional care, as part of the teams’ work process and under the responsibility of municipal management, based on the principles of comprehensive PHC and applicable in different contexts.

The importance of evaluating implementation at the municipal level lies in the fact that this is the management level responsible for the direct execution of actions and the organization of the care network. Understanding how different municipalities, with varying capacities and socioeconomic contexts, interpret and operationalize national FN guidelines is essential for identifying institutional bottlenecks. Defining the degree of implementation (DI) is a priority step, since the effectiveness of interventions cannot be ensured without first determining how local organizational characteristics favor or limit the dynamics of nutritional care^9^.

From this perspective, the objective of this study was to evaluate the implementation of nutritional care within the PHC system in Brazilian municipalities and to identify the association between the DI and aspects of the sociodemographic and political-organizational context.

## METHODS

This is an evaluative study with a quantitative approach, specifically an implementation analysis, which aims to determine the DI in different contexts and identify the influence of the context on the DI^10,11^.

The evaluation model was defined by consensus among expert groups in 2017, guided by the responsibility of municipal managers to ensure the structural and organizational conditions and the provision of nutritional care within the framework of comprehensive PHC. The evaluation model, including details on the consensus, is published in Machado et al.^12^ The judgment analysis matrix, consisting of two dimensions, six subdimensions, 17 indicators, 33 measures, and their respective parameters, is presented in the Chart.

**Chart:** Analysis and evaluation matrix for nutritional care in PHC, including the parameters used in the measures and indicators.

Subdimension	Indicators	Measures	Parameters	Aggregation^a^	Source
**DIMENSION 1 – MANAGEMENT**					
1.1 Infrastructure	1.1.1 Adequacy of physical space at the UBS for health care	Percentage of UBS with a room for group activities	3rd quartile: good Interval: fair 1st quartile: poor^b^	Both good (10) Most are fair (5) Both poor (0)	PMAQ
		Percentage of UBS with an examination room equipped with a computer and internet access for clinical care	3rd quartile: good Interval: fair 1st quartile: poor^b^		PMAQ
	1.1.2 Availability of equipment for FN interventions	Percentage of UBS with equipment available and in working condition for food and nutrition interventions (150/200 kg adult anthropometric scale, pediatric scale, measuring tape, anthropometric ruler)	100%: good < 100%: poor	Good (10) Poor (0)	PMAQ
	1.1.3 Availability of Supplies for Nutritional Care	Percentage of UBS with available nutritional supplements (oral rehydration salts, ferrous sulfate, folic acid)	100%: good < 100%: poor	Good (10) Poor (0)	PMAQ
1.2 Human Resources	1.2.1 Adequacy of PHC Staff	Estimated population coverage by primary care teams (ESF and parameterized teams)^c^	Up to 10,000 inhabitants = 100%: good Between 10,000 and 50,000 inhabitants = 80%: good Over 50,000 inhabitants = 60%: good	Both good (10) Most are fair (5) Both poor (0)	CNES/IBGE
		Percentage of complete minimum teams in the municipality	100%: good < 100%: poor		CNES/PMAQ
	1.2.2 Support from other professional categories for nutrition action programs	Percentage of UBS that have health professionals in addition to the minimum team (support from a nutritionist and at least one other professional, such as a social worker, psychologist, or physical educator)	3rd quartile: good Intermediate: fair 1st quartile: poor^b^	Both good (10) Most are fair (5) Both poor (0)	PMAQ
		Percentage of teams that have matrix support (eMulti or CAPS or network specialists or health surveillance or health academy hubs)	3rd quartile: good Interval: average 1st quartile: poor^b^		PMAQ
1.3 Organization and Standardization	1.3.1 Conditions for Access to Health Care	Percentage of UBS offering care during lunch hours or extended evening hours	50%: good < 50%: poor	3 “good” = good (10) 2 “good” = fair (5) ≤ 1 “good” = poor (0)	PMAQ
		Percentage of teams that schedule appointments on any day and at any time, either in person or by phone	100%: good < 100%: poor		PMAQ
		Percentage of teams that conduct risk and vulnerability assessments of users upon admission	≥ 90%: good 89% to 75%: fair < 75%: poor		PMAQ
	1.3.2 Referral flows to different points of care	Percentage of teams that receive assistance from other professionals in the network to resolve cases considered complex	≥ 90%: good 89% to 75%: fair < 75%: poor	Both good (10) Mostly fair (5) Both poor (0)	PMAQ
		Percentage of teams that refer obese patients to specialized services	≥ 90%: good 89% to 75%: fair < 75%: poor		
		Percentage of teams that receive feedback on evaluations conducted by specialists in the network	≥ 90%: good 89% to 75%: fair < 75%: poor		PMAQ
	1.3.3. Standardization of Health Care	Percentage of teams that use risk stratification protocols for (systemic hypertension, diabetes mellitus, children under 2 years of age)	≥ 90%: good 89% to 75%: fair < 75%: poor	Good (10) Fair (5) Poor (0)	PMAQ
	1.3.4 Availability of Information for Nutritional Care	Regular updates to the food consumption information systems for all life stages (Sisvan *Web*) over the last four-year period (2011–2014)	Yes: good No: poor	3 “good” = good (10) 2 “good” = fair (5) 1 or 0 “good” = poor (0)	Sisvan *Web*
		Regular updates to the information systems on nutritional status across all life stages (Sisvan Web) over the last four-year period (2010–2014)	Yes: good No: poor		Sisvan *Web*
		Percentage of children under five with nutritional status records in Sisvan *Web*^d^	≥ 80%: good < 80%: poor		Sisvan Web/IBGE
**DIMENSION 2 – HEALTH CARE**					
2.1 Food and Nutrition Surveillance	2.1.1 Continuous Monitoring of Nutritional Status and Food Consumption	Percentage of teams conducting food and nutrition surveillance activities	100%: good < 100%: poor	3 “good” = good (10) 2 “good” = fair (5) ≤ 1 “good” = poor (0)	PMAQ
		Percentage of teams that monitor breastfeeding rates and healthy complementary feeding	100%: good < 100%: poor		PMAQ
		Percentage of teams that conduct nutritional assessments at school	3rd quartile: good Interval: fair 1st quartile: poor^b^		PMAQ
	2.1.2 Local health planning	Percentage of teams that use maps showing the territory	100%: good < 100%: poor	Both good (10) Most are fair (5) Both poor (0)	PMAQ
		Percentage of teams that monitor and analyze health indicators and information	≥ 90%: good 89% to 75%: fair < 75%: poor		PMAQ
2.2 Promotion of Adequate and Healthy Nutrition	2.2.1 Development of Food and Nutrition Education Activities	Percentage of teams conducting food and nutrition education activities on breastfeeding and healthy eating	100%: good < 100%: poor	Both good (10) Most are fair (5) Both poor (0)	PMAQ
		Percentage of teams that invite obese users to participate in health promotion groups focused on healthy eating and physical activity	100%: good < 100%: poor		PMAQ
	2.2.2 Intersectoral coordination in the community	Percentage of teams that carry out health promotion activities in schools with	3rd quartile: good Interv: fair 1st quartile: poor^b^	Both good (10) Most are fair (5) Both poor (0)	PMAQ
		Percentage of schools implementing food and nutrition security initiatives and promoting adequate nutrition in schools	3rd quartile: good Interval: fair 1st quartile: poor^b^		PMAQ
2.3 Control and Monitoring of Nutritional Disorders	2.3.1 Requesting Diagnostic Tests in PHC	Percentage of teams requesting clinical tests within the healthcare network (creatinine, lipid profile, glycated hemoglobin, fasting blood glucose during prenatal care, hemoglobin and hematocrit levels during prenatal care)	≥ 90%: good 89% to 75%: fair < 75%: poor	Good (10) Fair (5) Poor (0)	PMAQ
	2.3.2 Organization of care according to nutritional risk	Percentage of teams that organize their schedules according to the classified risk for patients (pregnant women, patients with hypertension, patients with diabetes, obese patients, children up to 2 years of age)	≥ 90%: good 89% to 75%: fair < 75%: poor	3 “good” = good (10) 2 “good” = fair (5) ≤ 1 “good” = poor (0)	PMAQ
		Percentage of teams that schedule follow-up appointments for patients diagnosed with obesity	≥ 90%: good 89% to 75%: fair < 75%: poor		PMAQ
		Percentage of teams that actively screen for children with developmental delays and low weight and maintain records of developmental and nutritional status information	100%: good < 100%: poor		PMAQ
	2.3.3 Interdisciplinary and Multidisciplinary Care	Percentage of teams that enlist the support of other professionals to monitor users with obesity	3rd quartile: good Intervention: fair 1st quartile: poor^b^	Both good (10) Most are fair (5) Both poor (0)	PMAQ
		Percentage of teams that carry out shared activities with the schedule organized jointly by all professionals on the team	≥ 90%: good 89% to 75%: fair < 75%: poor		PMAQ
	2.3.4 Care Coordination	Percentage of teams that maintain records of patients with hypertension, diabetes, obesity, and high-risk pregnant women who are referred to other care facilities	≥ 90%: good 89% to 75%: fair < 75%: poor	Both good (10) Most are fair (5) Both poor (0)	PMAQ
		Percentage of teams that manage cases requiring additional attention	≥ 90%: good 89% to 75%: fair < 75%: poor		PMAQ

Interv.: interval between the 1st and 3rd quartiles; FN: food and nutrition; PHC: Primary Health Care; ESF: Estratégia Saúde da Família (Family Health Strategy); UBS: unidade básica de saúde (Basic Health Unit); PMAQ: Programa de Melhoria do Acesso e da Qualidade da Atenção Básica (Program for Improving Access and Quality of Primary Care); eMulti: Equipes Multiprofissionais na Atenção Primária à Saúde (Multidisciplinary Teams in Primary Health Care); CAPS: Centro de Atenção Psicossocial (Psychosocial Care Center); IBGE: Instituto Brasileiro de Geografia e Estatística (Brazilian Institute of Geography and Statistics); CNES: Cadastro Nacional de Estabelecimentos de Saúde (National Registry of Health Facilities); Sisvan: Sistema Nacional de Vigilância Alimentar e Nutricional (National Food and Nutrition Surveillance System).

^a^ The measures were aggregated to calculate the score for each indicator.

^b^ Parameters defined according to the quartile distribution for each population size stratum.

^c^ Parameter defined in accordance with Ordinance No. 1,007/2010 for the classification of financial incentives and Ordinance No. 2,355/2013 for the recommended average of 3,000 inhabitants per team. Both published by the Ministry of Health.

^d^ The national average vaccination coverage of 83% was used.

Secondary data from various national information systems were used, such as the Ministry of Health’s website to access the results of the second cycle of the external evaluation of the Programa de Melhoria do Acesso e da Qualidade da Atenção Básica (PMAQ-AB – Program for Improving Access and Quality of Primary Care) for 2013–2014, the Cadastro Nacional de Estabelecimentos de Saúde (CNES – National Registry of Health Facilities) to verify unidades básicas de saúde (UBS – primary health care units), the Instituto Brasileiro de Geografia e Estatística (IBGE – Brazilian Institute of Geography and Statistics) for municipal population sizes (2010), and the Sistema Nacional de Vigilância Alimentar e Nutricional (Sisvan – National Food and Nutrition Surveillance System) (2011–2014) to verify updated data on nutritional status and food consumption. For contextual variables, we additionally consulted databases from the United Nations Development Program (UNDP Brazil) and the stratification of Brazilian municipalities according to conditions influencing health management, as provided by the Núcleo de Extensão e Pesquisa em Avaliação em Saúde (Center for Extension and Research in Health Evaluation) at the Universidade Federal de Santa Catarina^13^.

Given the voluntary nature of participation in the PMAQ-AB and the potential for municipal managers to strategically select higher-performing facilities, the eligibility criterion adopted was to include only municipalities with participation of 80% or more of their total UBS and teams registered in the CNES. This methodological decision aimed to reduce selection bias and ensure greater internal consistency in the representation of the municipal analysis, as used in a previously published evaluation study^14^. The decision to use data from the second cycle of the PMAQ is justified by the completeness of information related to nutritional care, which is the focus of the evaluation.

The measures and indicators were assessed according to the parameters defined in the judgment analysis matrix (Chart). To ensure comparability among homogeneous groups of municipalities, some measures were assessed according to quartile distribution by municipal size. The value judgment (VJ) for the subdimensions, dimensions, and DI was determined by the percentage difference between the maximum score (MS) and the observed score (OS), using the formula VJ = (∑ OS / ∑ MS) × 100. The indicators, subdimensions, and dimensions were classified as “good” when they reached values above 66.6% of the MS, as “poor” when they reached up to 33.3%, and as “fair” in all other cases. A weight of 2 was assigned to the OS in the *Care* dimension, due to its closer alignment with the evaluation objective.

The classification of the DI for nutritional care in PHC followed the methodology proposed by Vieira da Silva et al.^15^, in which DI categories were determined based on tertile distribution cutoff points, using the following criteria: (1) Incipient: values > 0 and < 33.3%; (2) Intermediate: values > 33.3% and < 66.6%; (3) Advanced: values > 66.6%.

The descriptive analysis examined the distribution of municipalities according to the VJ of the dimensions, subdimensions, and indicators, as well as according to the DI categories and context variables. A chi-square test was performed to identify the factors associated with the implementation of nutritional care and to define the eligible variables (p < 0.20) for the regression model.

The independent variables used were: Municipal Size (> 100,000 inhabitants / between 25,000 and 100,000 / < 25,000); Region (North/Northeast/Southeast/South/Central-West); Municipal Human Development Index (mHDI) (tertil); gross domestic product (GDP) *per capita* (tertil); proportion of the population under five years of age (tertil) for Block 1 – Socioeconomic and Demographic Aspects. And the variables: Municipal strata according to capacity for health management; Receipt of funding for nutrition interventions (No/Yes); Registration of a manager for nutrition interventions (No/Yes); Support from a dietitian in primary health care (No/Yes); and ESF coverage comprised Block 2 – Political and Organizational Aspects.

Regarding municipal strata based on capacity for health management, this classification is based on the combination of demographic characteristics (population density and urbanization rate), capacity for funding (GDP *per capita*), and the population’s purchasing power (percentage in extreme poverty and health insurance coverage), which together define the conditions for managing the local health system into three classes: favorable, fair, or unfavorable. The three categories, combined with the municipalities’ population size, classify them into seven strata^13^, namely:

- Small, unfavorable: municipalities with up to 25,000 inhabitants and unfavorable factors for management;
- Small, fair: municipalities with up to 25,000 inhabitants and fair factors influencing management;
- Small, favorable: municipalities with up to 25,000 inhabitants and favorable factors for management;
- Medium, unfavorable: municipalities with 25,000 to 100,000 inhabitants and factors unfavorable for management;
- Medium, neutral: municipalities with 25,000 to 100,000 inhabitants and neutral factors affecting governance;
- Medium favorable: municipalities with 25,000 to 100,000 residents and influential figures favorable to management;
- Large: municipalities with more than 100,000 inhabitants, regardless of the factors.

The outcome was dichotomized into *“implemented* (1)*”* and *“not implemented* (0)*”* for association tests with the two blocks of independent variables. As a measure of association, odds ratios (OR) were calculated, along with their respective 95% confidence intervals (95% CI), using multivariate logistic regression.

Two regression models were created, adjusted for the variables in block 1 and block 2, using a probability test in each block to identify subgroups of categories with the highest and lowest likelihood of implementing nutritional care in the municipalities. Model diagnosis was performed using the “goodness-of-fit” test*.* Categories with a prevalence of the outcome below 1% were removed from the variables “municipal size” and “municipal strata,” based on their performance in the regression models.

The data were processed in a spreadsheet using Microsoft Office Excel^®^ (Microsoft Corp., United States) and then transferred to the statistical software Stata^®^ 13.0 (Stata Corp., College Station, United States). In accordance with the methodological steps involving consensus and stakeholder engagement^12^, this study was approved by the Ethics Committee of the Universidade Federal de Santa Catarina, in compliance with Resolution No. 466/2012 of the Ministry of Health, under approval number 2.047.153/2017.

## RESULTS

A total of 4,064 Brazilian municipalities were evaluated, representing 73% of all Brazilian municipalities in 2014. The sample exhibits a homogeneous distribution, with a representation rate above 50% in most states, except for Maranhão (15.7%), Acre (31.8%), and Roraima (40.0%).

Nutritional care in PHC shows varying levels of implementation across the analyzed municipalities. The majority (57.7%) are at an intermediate stage, while 30.7% are at an incipient level and only 11.6% at an advanced level. Regionally, Santa Catarina (24.3%) and Rio Grande do Sul (23.5%) have the highest percentage of municipalities with advanced implementation. In contrast, nine states, including Acre, Amazonas, Roraima, Rondônia, Pará, Alagoas, Mato Grosso do Sul, Espírito Santo, and Rio de Janeiro, had no municipalities with advanced implementation of nutritional care in PHC (Figure).

**Figure:**
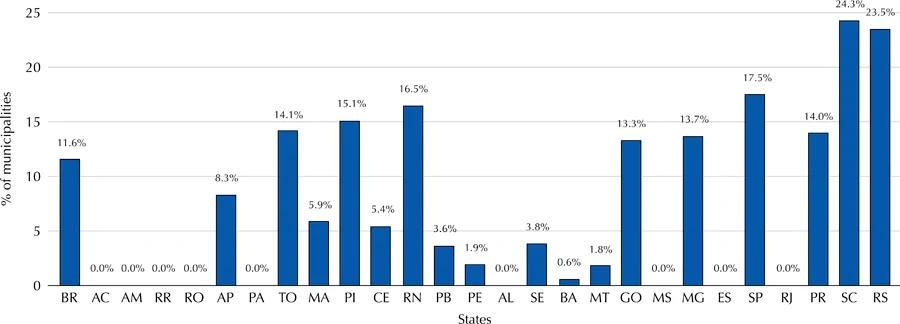
Percentage distribution of municipalities with nutritional care in Primary Health Care implemented at an advanced level, relative to the total number of municipalities evaluated, by state (n = 4,064).

BR: Brazil; AC: Acre; AM: Amazonas; RR: Roraima; RO: Rondônia; AP: Amapá; PA: Pará; TO: Tocantins; MA: Maranhão; PI: Piauí; CE: Ceará; RN: Rio Grande do Norte; PB: Paraíba; PE: Pernambuco; AL: Alagoas; SE: Sergipe; BA: Bahia; MT: Mato Grosso; GO: Goiás; MS: Mato Grosso do Sul; MG: Minas Gerais; ES: Espírito Santo; SP: São Paulo; RJ: Rio de Janeiro; PR: Paraná; SC: Santa Catarina; RS: Rio Grande do Sul.

Comparing the two dimensions of analysis in the evaluation matrix—management and care—the results show a poorer performance in the *Management* dimension. This dimension assesses the assurance of structural and organizational conditions for the provision of FN and found that only 8.2% of municipalities were classified as “good.” When analyzing the subdimensions, aspects related to *Infrastructure* and *Human Resources* were considered satisfactory in just over one-third of the municipalities—36.2% and 33.7%, respectively. Less than 1% met the necessary criteria for a “good” rating in the *Organization and Standardization* subdimension (Table 1).

**Table 1:** Distribution of municipalities evaluated for the implementation of nutritional care in PHC according to the indicators, subdimensions, and value-judgment dimensions used (n = 4,064).

Dimension	Classification					
Subdimension	Poor		Fair		Good	
Indicators	n	%	n	%	n	%
**Management**	**1,280**	**31.5**	**2,451**	**60.3**	**333**	**8.2**
Infrastructure	879	21.6	1,715	42.2	1,470	36.2
Suitability of physical space	759	18.7	2,628	64.7	677	16.7
Equipment Availability	1,135	27.9	-	-	2,929	72.1
Availability of inputs	2,674	65.8	-	-	1,390	34.2
Human Resources	1,016	25.0	1,678	41.3	1,370	33.7
Staffing levels	779	19.2	2,072	51.0	1,213	29.8
Support from other professional categories	698	17.2	2,692	66.2	674	16.6
Organization and Standardization	2,741	67.5	1,288	31.6	35	0.9
Healthcare Access Conditions	2,639	64.9	1,311	32.3	114	2.8
Referral flows	1,505	37.0	2,128	52.4	431	10.6
Standardization of health care	2,477	61.0	316	7.7	1,271	31.3
Availability of information	3,669	90.2	368	9.1	27	0.7
**Care**	**1,370**	**33.7**	**1,894**	**46.6**	**800**	**19.7**
Food and Nutrition Surveillance	975	24.0	979	24.1	2,110	51.9
Ongoing monitoring	1,273	31.3	1,465	36.1	1,326	32.6
Local health planning	334	8.2	1,511	37.2	2,219	54.6
Promotion of Adequate and Healthy Nutrition	2,038	50.2	908	22.3	1,118	27.5
Development of EAN Activities	2,289	56.3	1,156	28.4	619	15.2
Cross-sectoral coordination at the local level	763	18.8	1,923	47.3	1,378	33.9
Control and Monitoring of Nutritional Disorders	1,473	36.3	2,163	53.2	428	10.5
Requests for diagnostic tests in PHC	1,094	26.9	499	12.3	2,471	60.8
Organization of care based on nutritional risk	3,205	78.9	614	15.1	245	6.0
Interdisciplinary and Multidisciplinary Care	730	18.0	2,687	66.1	647	15.9
Care Coordination	2,132	52.5	1,580	38.9	352	8.7

EAN: food and nutrition education; PHC: Primary Health Care.

When analyzing each subdimension separately, the “Availability of equipment for nutrition education activities” indicator stands out positively in *the Infrastructure category*, with most municipalities achieving the best result (72.1%). In contrast, “Suitability of physical space” was rated as “fair” in most municipalities (64.7%), while “Availability of supplies” presented the most critical situation, being rated as “poor” in 65.8% of the sample.

In the *Human Resources* subdimension*,* more than half of the municipalities scored “fair” on the indicators “Staffing Levels” (51%) and “Support from Other Professional Categories for Public Health Actions” (66.2%).

Among the indicators that make up the *Organization and Standardization* subdimension are the worst results for aspects of municipal management. More than 60% of the municipalities are classified as “poor” for “Conditions for Access to Health Care” (64.9%) and “Standardization of Care” (61%). Moreover, 90.2% of the evaluated municipalities performed “poorly” on the “Availability of Information for Nutritional Care” indicator.

The *Care* dimension, in turn, showed that 19.7% of municipalities performed well. This dimension evaluates aspects related to the organization of nutritional care provided by PHC teams. When comparing the subdimensions within the Care block, the most positive result was in *Food and Nutrition Surveillance*, with 51.9% of municipalities rated as “good.” In contrast, 50.2% of municipalities were classified as “poor” in the subdimension *“Promotion of Adequate and Healthy Diet*,” and in *“Control and Monitoring of Nutritional Disorders*,” few municipalities were rated “good” (10.5%).

Focusing on the *Food and Nutrition Surveillance* subdimension, the positive highlight was the “Local Health Planning” indicator, with 54.6% of municipalities rated as “good.” For “Continuous Monitoring of Nutritional Status and Food Consumption,” there was an even distribution of municipalities across the three evaluation criteria.

In the area *of Promotion of Adequate and Healthy Diet,* the worst rating was for the indicator “Development of Food and Nutrition Education Activities,” with 56.3% of municipalities rated as “poor” in this regard. The second indicator, which addressed “Inter-sectoral Coordination at the Local Level,” fared slightly better, with 47.3% of municipalities rated as “fair” and 18.8% as “poor.”

Despite the unsatisfactory results for aspects related to the *Control and Monitoring of Nutritional Disorders* subdimension*,* when the indicators are evaluated separately, the “Request for Diagnostic Tests in PHC” stands out as the indicator with the highest percentage of municipalities rated “good” (60.8%). In contrast, the indicators “Organization of care according to nutritional risk” and “Care Coordination” had the lowest percentages classified as “good” in the management dimension, at 6.0% and 8.7%, respectively (Table 1).

In the contextual analysis, apart from the factors *“Receipt of funds for FN initiatives* (2011–2013)” and *“Registration of a manager for FN initiatives*,” the remaining variables showed a statistically significant association with the GI for nutritional care (p < 0.001) (Table 2). Municipalities with fewer than 25,000 inhabitants had a 14.7% rate of advanced DI for nutritional care, and no municipality with more than 100,000 inhabitants was classified at this same level of implementation.

**Table 2:** Distribution of municipalities by degree of implementation and contextual characteristics (n = 4,064).

Variable	Total	Level of implementation of nutritional care			
		Incipient	Intermediate	Advanced	p-value^b^
**Context**	**n (%)**	**% (95% CI)**	**% (95% CI)**	**% (95% CI)**
Brazil	4,064 (100)	30.7	57.7	11.6	
*Block 1 - Socioeconomic and Demographic Aspects*					
Municipal Size					< 0.001
> 100,000	182 (4.5)	53.8 (46.5–61.1)	46.2 (38.8–53.5)	-	
Between 25,000 and 100,000	742 (18.3)	51.6 (48.0–50.5)	46.9 (43.3–50.5)	1.5 (0.6–2.3)	
< 25,000 residents	3,140 (77.2)	24.4 (22.9–25.9)	60.9 (59.2–62.6)	14.7 (13.4–15.9)	
Region					< 0.001
North	269 (6.6)	56.5 (50.5–62.5)	38.3 (32.4–44.1)	5.2 (2.5–7.9)	
Northeast	1,262 (31.1)	35.1 (32.5–37.7)	59.4 (56.6–62.1)	5.5 (4.3–6.8)	
Southeast	1,239 (30.5)	23.7 (21.4–26.1)	62.6 (59.8–65.2)	13.7 (11.8–15.6)	
South	899 (22.1)	21.8 (19.1–24.5)	57.6 (54.4–60.5)	20.6 (17.9–23.2)	
Central-West	395 (9.7)	41.3 (36.4–46.1)	50.6 (45.7–55.6)	8.1 (5.4–10.8)	
HDI^c^					< 0.001
0.418 to 0.625	1,358 (33.4)	35.8 (33.2–38.3)	57.6 (55.0–60.3)	6.6 (5.2–7.9)	
0.626 to 0.704	1,373 (33.8)	29.6 (27.2–32.0)	57.3 (54.7–60.0)	13.1 (11.3–14.9)	
0.705 to 0.862	1.333 (32.8)	26.7 (24.3–29.1)	58.1 (55.5–60.8)	15.2 (13.2–17.1)	
GDP *per capita*^c^					< 0.001
≥ R$ 2,782.87	1,355 (33.3)	33.8 (31.2–36.2)	58.9 (56.3–61.5)	7.3 (5.9–8.8)	
≥ R$ 6,462.94	1,355 (33.4)	30.6 (28.2–33.1)	57.7 (55.1–60.3)	11.7 (10.0–13.4)	
≥ R$ 13,436.55	1,354 (33.3)	27.8 (25.4–30.2)	56.5 (53.9–59.1)	15.7 (13.8–17.7)	
Proportion of the population under 5 years of age^c^					< 0.001
≥ 2.3%	1,355 (33.3)	20.9 (18.7–23.1)	60.0 (57.4–62.6)	19.1 (17.0–21.2)	
≥ 6.5%	1,355 (33.4)	31.0 (28.5–33.5)	59.1 (56.5–61.7)	9.9 (8.2–11.5)	
≥ 7.7%	1,354 (33.3)	40.3 (37.6–42.9)	54.0 (51.3–56.6)	5.7 (4.5–7.0)	
*Block 2 - Political and Organizational Aspects*					
Municipal strata according to capacity for health management^a^					< 0.001
Large	172 (4.2)	52.9 (45.4–60.4)	47.1 (39.5–54.6)	-	
Moderately favorable	268 (6.6)	41.8 (35.8–47.7)	56.0 (50.0–61.9)	2.2 (0.1–4.0)	
Average	245 (6.0)	55.6 (49.2–61.8)	42.8 (36.6–49.1)	1.6 (0.1–3.2)	
Unfavorable average	176 (4.3)	61.9 (54.7–69.2)	38.1 (30.8–45.3)	-	
Slightly favorable	466 (11.5)	23.2 (19.3–27.0)	60.3 (55.8–64.7)	16.5 (13.1–19.9)	
Small, average	1,495 (36.8)	22.7 (20.6–24.9)	60.6 (58.1–63.1)	16.7 (14.8–18.5)	
Small, unfavorable	1,241 (30.6)	28.3 (25.8–30.8)	60.8 (58.1–63.5)	10.9 (9.1–12.6)	
Funding received for FN initiatives (2011–2013)					0.072
No	88 (2.2)	22.7 (13.8–31.6)	59.1 (48.6–69.6)	18.2 (10.0–26.4)	
Yes	3,976 (97.8)	30.9 (29.4–32.3)	57.7 (56.1–59.2)	11.4 (10.4–12.4)	
Manager registration for FN initiatives					0.150
No	2,691 (66.2)	31.4 (29.6–33.2)	56.7 (54.7–58.5)	11.9 (10.7–13.2)	
Yes	1,373 (33.8)	29.4 (26.9–31.8)	59.8 (57.2–62.4)	10.8 (9.2–12.5)	
Support from a dietitian in primary care				< 0.001	
No	2,442 (60.1)	36.8 (34.9–38.8)	55.0 (53.1–57.0)	8.2 (7.0–9.2)	
Yes	1,622 (39.9)	21.5 (19.5–23.5)	61.7 (59.4–64.1)	16.8 (15.0–18.7)	
ESF coverage					< 0.001
Up to 75.0%	565 (13.9)	46.3 (42.1–50.3)	47.4 (43.3–51.6)	6.3 (4.3–8.4)	
Between 75.0% and 99.9%	484 (11.9)	45.7 (41.2–50.1)	49.8 (45.3–54.3)	4.5 (2.7–6.4)	
100%	3,015 (74.2)	25.4 (23.8–26.9)	60.9 (59.2–62.6)	13.7 (12.5–14.9)	

mHDI: Municipal Human Development Index; GDP: gross domestic product; FN: food and nutrition; PHC: Primary Health Care; ESF: Estratégia Saúde da Família (Family Health Strategy); 95% CI: 95% confidence interval.

^a^ Proposed municipal stratification based on capacity for health management according to Calvo et al.^13^

^b^ Chi-square test.

^c^ Categories referring to the sample distribution tertile.

The South and Southeast regions have the highest percentages of municipalities with nutritional care implemented at an advanced stage, at 20.6% and 13.7%, respectively. In contrast, the North and Central-West regions have the highest proportions of incipient implementation, with rates of 56.5% and 41.3%, respectively. In the Northeast, the intermediate stage predominates in 59.4% of the municipalities evaluated.

When analyzing the DI of nutritional care and its relationship with the mHDI and GDP *per capita* values, a directly proportional relationship with the outcome is observed, with higher prevalences of advanced implementation as the mHDI and GDP *per capita* increase. In contrast, the proportion of the population under five years of age was inversely proportional to the DI; the lower the proportion of children in the municipality, the higher the DI for nutritional care (19.1%).

Among municipal strata, based on management capacity, municipalities classified as “small-favorable” and “small-fair” showed the highest prevalence of advanced nutritional care GI, at approximately 16.5%. Municipalities that have the support of a dietitian in PHC and 100% coverage by the ESF have more than double the implementation rate of nutritional care (Table 2).

Table 3 presents the crude and multivariate regression analysis models. Socioeconomic and demographic factors showed a strong association with the implementation of nutritional care in the crude analysis. Municipalities with fewer than 25,000 inhabitants and a lower proportion of children aged five years or younger showed higher prevalence of nutritional care implementation after adjusting for the variables. Although the variables “region” and “municipal HDI” were no longer associated after adjustment, the categories “Southern region” and “municipal HDI greater than 0.705” retained their association.

**Table 3:** Association between contextual characteristics and the implementation of nutritional care in primary health care (n = 4,064).

Variable	Nutritional care implemented			
	Crude		Model	
Context	OR (95% CI)	p-value	OR (95% CI)	p-value
*Block 1 - Socioeconomic and demographic aspects*^a^				
Municipal size		< 0.001		< 0.001
≥ 25,000 inhabitants	1.00		1.00	
< 25,000 inhabitants	14.25 (7.80–26.03)		14.11 (7.65–26.02)	
Region		< 0.001		0.996
North	1.00		1.00	
Northeast	1.07 (0.59–1.93)		0.76 (0.39–1.47)	
Southeast	2.90 (1.65–5.08)		1.55 (0.82–2.92)	
South	4.72 (2.69–8.28)		1.98 (1.03–3.81)	
Central-West	1.61 (0.84–3.07)		0.87 (0.43–1.74)	
HDIm^d^		< 0.001		0.143
0.418 to 0.625	1.00		1.00	
0.626 to 0.704	2.15 (1.65–2.81)		1.26 (0.81–1.94)	
0.705 to 0.862	2.55 (1.96–3.31)		1.30 (0.79–2.13)	
GDP *per capita*^d^		< 0.001		0.024
≥ R$ 2,782.87	1.00		1.00	
≥ R$ 6,462.94	1.66 (1.27–2.15)		0.69 (0.45–1.04)	
≥ R$ 13,436.55	2.34 (1.82–30.1)		0.94 (0.60–1.47)	
Proportion of the population under 5 years of age^d^		< 0.001		< 0.001
≥ 2.3%	1.00		1.00	
≥ 6.5%	0.46 (0.37–0.58)		0.65 (0.51–0.84)	
≥ 7.7%	0.26 (0.20–0.34)		0.53 (0.38–0.76)	
*Block 2 - Political and Organizational Aspects*^b^				
Municipal strata according to capacity for health management^c^		< 0.001		< 0.001
Moderately favorable	1.00		1.00	
Moderate	1.20 (0.34–1.30)		1.08 (0.30–3.92)	
Small, favorable	9.86 (4.26–22.84)		8.77 (3.71–20.73)	
Slightly neutral	14.46 (6.38–32.72)		13.07 (5.62–30.40)	
Small, unfavorable	8.83 (3.87–20.15)		7.91 (3.36–18.63)	
Funding received for FN initiatives (2011–2013)		0.068		
No	1.00			
Yes	0.58 (0.34–1.01)			
Manager registration for FN initiatives		0.292		
No	1.00			
Yes	0.90 (0.73–1.10)			
Support from a dietitian in primary care		< 0.001		< 0.001
No	1.00		1.00	
Yes	2.29 (1.89–2.79)		2.40 (1.97–2.94)	
ESF coverage		< 0.001		0.005
Up to 75.0%	1.00		1.00	
Between 75.0% and 99.9%	0.70 (0.41–1.21)		0.46 (0.28–0.87)	
100%	2.33 (1.64–3.32)		1.11 (0.76–1.64)	

MHDI: Municipal Human Development Index; GDP: gross domestic product; FN: food and nutrition; PHC: Primary Health Care; ESF: Estratégia Saúde da Família (Family Health Strategy); OR: odds ratio; 95% CI: 95% confidence interval.

^a^ Model 1 included variables with p < 0.05 in the crude analysis, using the variables from block 1 adjusted for one another.

^b^ Model 2 included variables with p < 0.05 in the crude analysis, using the variables from block 2 adjusted for one another.

^c^ Proposed municipal stratification according to Calvo et al.^13^.

^d^ Categories referring to the sample distribution tertiles.

When a likelihood test was performed among the variables that remained statistically significant after adjusting for each block (p < 0.001), municipalities with more than 25,000 inhabitants, lower GDP *per capita* (1st tertile ≥ R$ 2,782.87), and a higher proportion of children under five (3rd tertile ≥ 7.7%) have less than a 0.1% chance of advanced implementation of nutritional care. Municipalities with fewer than 25,000 inhabitants, higher GDP *per capita* (3rd tertile ≥ R$ 13,436.55), and a lower proportion of children under five (1st tertile ≥ 2.3%) have a 25.8% chance of implementing nutritional care (data not shown in the table).

In contrast, municipalities without support from a dietitian in PHC, in the favorable medium stratum, with up to 75% ESF coverage, and which received funding for nutritional care initiatives, have a 2.3% chance of advanced implementation of nutritional care. Those with nutritionist support in PHC, in the “small regular” stratum, with 100% ESF coverage, and which did not receive funding for FN initiatives have a 36.0% chance of implementing nutritional care (data not shown in the table).

## DISCUSSION

The implementation of nutritional care in Brazilian PHC is predominantly intermediate or incipient, with the greatest weaknesses concentrated in the area of municipal management. The study reveals that, while food and nutrition surveillance (FNS) has made progress, structural organization and health promotion initiatives still face critical barriers to implementation. The success of implementation was found to be strongly influenced by better socioeconomic indicators, a small population size, the strategic presence of a dietitian in PHC, and ESF coverage.

In the Management dimension, municipalities showed low compliance with the criteria listed for *Infrastructure*, *Human Resources*, and aspects of *Organization and Regulation*, conditions that are indispensable to other care pillars of PHC. This scenario reinforces the fact that expanding access and the structuring of PHC services in the country require addressing chronic managerial challenges to effectively transform the health care model in Brazil^16^.

Among these challenges, within the *Infrastructure* subdimension, there is a marked inconsistency characterized by high equipment availability in contrast to a shortage of supplies and inadequate physical spaces. This scenario reflects the federal-led model in effect during the period (such as the PMAQ-AB), which prioritized the allocation of funds for the acquisition of capital assets. However, this model encountered barriers related to logistical planning capacity and municipal funding for the maintenance of basic supplies^14^.

Most of the municipalities analyzed lacked oral rehydration salts, ferrous sulfate, and folic acid—essential supplies for nutritional care that are included in the list of essential medicines in the Relação Nacional de Medicamentos (National Drug List)^17^. Regarding physical space, the measures employed indicated the need for at least one examination room equipped with a computer and internet access in health facilities. This infrastructure was essential for properly filling out the registration forms in Sisvan Web*,* as well as for consulting databases to use the information in planning.

These findings demonstrate that the mere possession of material resources is insufficient to consolidate nutritional care in PHC. The lack of adequate physical space in UBS compromises patient reception and nutritional care, while the lack of a steady supply of supplies limits the effectiveness of educational and surveillance initiatives, resulting in underutilized equipment and inconsistent care practices in the region^14^.

The negative result for the *Organization and Standardization* subdimension, particularly regarding the availability of information for nutritional care, is a critical finding. This indicator assessed the systematic updating of data on food consumption and nutritional status in Sisvan Web, as well as the monitoring of weight and height in children under five years of age. Although the guidelines of the PNAN and the Programa Bolsa Família (Bolsa Família Program) established this monitoring as a priority, the study’s results suggest that data collection was often limited to purposes related to financial transfers.

These findings may be explained by the lack of established protocols and the limited presence of nutritionists in PHC to perform nutritional assessments^18^. Furthermore, unlike the current situation, which is characterized by efforts to promote interoperability among information systems, the 2014 landscape was one of fragmentation, with Sisvan operating in isolation. These factors contribute to an administrative record that is disconnected from data analysis, care planning, or early intervention^19,20^, indicating weaknesses in municipal management’s ability to use the FNS strategically.

Finally, still within this subdimension, the low adequacy in the areas of health care access and standardization of care reveals barriers that directly compromise the service’s effectiveness. The limited availability of extended hours and difficulties in scheduling demonstrate that organizational access was still marked by structural rigidity. At the same time, the limited use of risk stratification protocols for hypertension, diabetes, and child health indicates that nutritional care operated reactively. Without standardization based on vulnerability, nutritional care loses its predictive capacity and ceases to function as a strategic pillar in the HCN^1^.

Regarding the *Care* dimension, it showed a slightly higher level of adequacy, although it was still limited to a small proportion of municipalities. However, a significant internal disparity is noted among the sub-dimensions: while the FNS emerges as the most well-established pillar in the daily practice of PHC, actions related to the Promoção da Alimentação Adequada e Saudável e o Controle (Promotion of Adequate and Healthy Nutrition) and the Acompanhamento dos Agravos Nutricionais (Control and Monitoring of Nutritional Disorders) reveal profound gaps. This imbalance suggests that, during the period analyzed, the teams’ capacity to monitor the population did not translate into practical health promotion interventions or specialized clinical management, indicating a predominantly diagnostic approach to care.

When examining the FNS subdimension in detail, it is observed that its superior performance is largely driven by advances in local planning. This result suggests that the logic of territorialization and the culture of monitoring health indicators, pillars of the ESF and encouraged by financial incentive programs, were more firmly established in the teams’ routines^4,5^. However, the monitoring of breastfeeding and school health initiatives, although regulated, face certain implementation challenges that may be linked to the fragmentation of care practices and the lack of specialized technical support to translate territorial diagnosis into direct clinical intervention^18^.

On the other hand, the subdimensions focused on the *Promotion of Adequate and Healthy Nutrition* and the *Control and Monitoring of Nutritional Disorders* reveal the greatest obstacles to effective care. The significant inadequacy in the development of Food and Nutrition Education activities suggests that, in 2014, promotion practices still faced difficulties in moving beyond purely prescriptive models^21^. It is important to emphasize that initiatives to promote adequate and healthy eating among mothers and children are fundamental to comprehensive health care and are supported by evidence regarding their potential to reverse low breastfeeding rates^22,23^.

This gap becomes even more evident in addressing nutritional disorders. While the capacity to order diagnostic tests performs satisfactorily, the organization of care using risk classification protocols or holding PHC accountable for cases referred to other care facilities ranks among the most critical issues in the *Care* dimension. This scenario indicates the persistence of a biomedical approach centered on disease identification, but one that seriously fails to manage the user’s therapeutic pathway in an orderly manner.

Some studies corroborate these findings, highlighting the challenge faced by PHC in establishing care pathways and organizing care sites in a coordinated manner that is appropriate to users’ health needs^24,25^. This lack of coordination reaffirms that surveillance, when isolated from clinical management and stratification protocols, is insufficient to bring about real change in the population’s epidemiological profile. This tendency toward a fragmented care model suggests the persistence of a selective PHC system^5^, in which access to diagnostic tests does not, by itself, guarantee comprehensive nutritional care.

Regarding contextual characteristics, municipalities with fewer than 25,000 inhabitants, located in the Southern region, with better social indicators (HDI and GDP *per capita*) and a lower proportion of children were identified as having conditions favorable to the implementation of nutritional care in PHC. Better evaluation results in smaller municipalities were also found in studies assessing oral health^26^, health planning management^27^, and work processes in primary health care^28^.

Regional disparities in the provision of nutritional care reflect historical structural inequalities in the Brazilian health system. The prevalence of advanced stages in the South and Southeast regions coincides with greater socioeconomic development and a concentration of infrastructure and a specialized workforce, particularly nutritionists^29,30^. In contrast, the high rates of incipient implementation in the North and Central-West regions highlight logistical challenges, such as vast territorial distances and poor connectivity to support the systems, as well as the difficulty in retaining professionals in remote areas. These factors limit the effectiveness of interventions beyond basic FNS records, exacerbating gaps in care in these regions^29,31^.

Regarding GDP and the mHDI, studies have shown that poorer social indicators are associated with higher rates of infant mortality and hospitalizations for conditions treatable through primary care^32^ and may be related to the limited implementation of nutritional care in PHC. Studying these complex mediating factors allows us to identify where and how interventions should be carried out, with the aim of reducing health inequities and pinpointing the areas where such interventions can have the greatest impact^33^.

The presence of a dietitian as a support professional in PHC and municipalities where 100% of the population is covered by the ESF were also found to be strongly associated with better implementation of nutritional care. The nutritionist is considered a key professional for providing cross-functional support to FN initiatives in PHC^34^ and is essential for integrating health promotion initiatives across all life stages, enhancing efforts related to healthy eating and food and nutritional security^35^.

Despite the allocation of specific funds for the implementation of nutritional care in PHC and the presence of coordinators for FN initiatives within municipal health departments, these conditions did not result in improved DI. A study on PHC in Brazil highlights that the main challenges to the implementation process are insufficient funding and professional training that is not guided by the comprehensive care model^36^.

## CONCLUSION

The study shows that the implementation of nutritional care in Brazilian PHC in 2014 was in its early stages, marked by significant advances in aspects of FNS but still limited by critical weaknesses in the management and coordination of care. The picture that emerges points to a “care paradox”: municipalities demonstrate technical competence in territorial planning and in performing diagnostic procedures, such as ordering tests, but systematically fail to deliver end-goal interventions, such as food and nutrition education and the clinical management of nutritional disorders.

The low DI of nutritional care in PHC, given the complexity of the country’s current contexts, does not diminish the contributions of PHC to the population’s health, nor its potential for reorganizing health services and establishing a new care model. On the other hand, these findings point to the need for continuous processes of evaluation and monitoring of interventions, improvement of care quality, and the integration of nutritional care as part of the care model. Identifying municipalities with a higher or lower likelihood of implementing nutritional care enables the planning of policies that promote greater equity in the distribution of health resources.

This reality suggests that federal incentives through quality improvement programs were effective in establishing a culture of monitoring and record-keeping, but insufficient to break away from the logic of selective PHC, which remains centered on biomedical and prescriptive models. The marked disconnect between data collection and clinical practice reaffirms the urgent need to strengthen the role of nutritionists in the community, not merely as occasional technical support, but as essential coordinators within the HCN, carrying out more comprehensive and decisive actions.

Finally, the results indicate that strengthening nutritional care within the SUS depends on overcoming fragmented actions, the isolation of information systems, and the rigidity of organizational models. It is hoped that the path toward technological integration initiated during the period analyzed will evolve into strategic information management, capable of transforming epidemiological data into guidelines for comprehensive care. Overcoming regional disparities and ensuring that the FNS translates into improved health therefore remain the main challenges for consolidating the PNAN in the current context.
