# Supplementary material for: Analysis of the implementation of nutritional care in the context of Primary Health Care
Source: Rev Saude Publica. 2026 Sep 9;60:e43. doi: 10.11606/s1518-8787.2026060006929 (PMC13623370; doi:10.11606/s1518-8787.2026060006929)
Supplement: Translation

# Translation: Análise de implantação da atenção nutricional no contexto da Atenção Primária à Saúde

**Authors:** Patrícia Maria de Oliveira Machado, Josimari Telino de Lacerda, Rafaela Souza Petrolini, Mick Lennon Machado

**Affiliations:** I Brasil Universidade Federal de Santa Catarina. Centro de Ciências da Saúde. Departamento de Saúde Pública. Florianópolis, SC, Brasil

**Keywords:** Avaliação em Saúde, Programas e Políticas de Nutrição e Alimentação, Pesquisa sobre Serviços de Saúde

## Abstract

**OBJETIVO:**

Avaliar a implantação da atenção nutricional no âmbito da Atenção Primária à Saúde em municípios brasileiros e identificar a associação do grau de implantação com aspectos do contexto sociodemográfico e político-organizacional.

**MÉTODOS:**

Trata-se de uma pesquisa avaliativa, com abordagem quantitativa, que considerou a responsabilidade do gestor municipal na garantia das condições e do provimento de ações, a partir da análise de 17 indicadores agrupados nas dimensões gestão e assistência. Os municípios foram classificados segundo o grau de implantação da atenção nutricional na Atenção Primária à Saúde pelo ajuizamento da pontuação observada em relação à pontuação máxima, utilizando critérios incipiente, intermediário e avançado. Analisou-se a influência do contexto sobre o grau de implantação, com as variáveis independentes agrupadas em dois blocos, utilizando-se regressão logística multivariada.

**RESULTADOS:**

Foram avaliados 4.064 municípios brasileiros, sendo observado grau de implantação avançado em 11,6% (n = 471). Os aspectos de gestão receberam piores resultados quando comparados aos de assistência. Os fatores de contexto associados ao melhor grau de implantação da atenção nutricional foram municípios com porte populacional menor de 25.000 habitantes, localizados na Região Sul, melhores indicadores sociais, menor proporção de crianças menores de cinco anos, 100% de cobertura da Estratégia Saúde da Família e apoio do nutricionista na Atenção Primária à Saúde.

**CONCLUSÃO:**

A implantação da atenção nutricional na Atenção Primária à Saúde brasileira foi considerada incipiente no período investigado. Apesar dos avanços significativos em relação à vigilância alimentar e nutricional, a atenção nutricional apresentou fragilidades importantes na gestão e na coordenação do cuidado. Reitera-se a necessidade de processos contínuos de avaliação e monitoramento das intervenções na Atenção Primária à Saúde, qualificação da assistência e inserção da atenção nutricional como parte do modelo assistencial.

## INTRODUÇÃO

A atenção nutricional é caracterizada como o conjunto de cuidados em alimentação e nutrição (AN) voltados à promoção, prevenção, diagnóstico e tratamento de agravos em saúde^1^. É reconhecida como um importante eixo assistencial e encontra na Atenção Primária à Saúde (APS) um espaço estratégico para a sua implantação e consolidação^2,3^.

A APS é considerada porta de entrada preferencial da Rede de Atenção à Saúde (RAS), pois apresenta alto grau de capilaridade e maior proximidade dos indivíduos e famílias nos territórios^4^. Nesse contexto, a APS do tipo abrangente ou integral difere da APS seletiva voltada para pacotes restritos de intervenções de baixo custo para grupos populacionais específicos e agravos isolados^5^. A APS abrangente ou integral tem como premissa as necessidades de saúde da população e a integralidade do cuidado, refletindo o seu potencial de enfrentamento da situação epidemiológica e nutricional atual^4^.

Apesar dos aspectos normativos que integram a atenção nutricional no sistema de saúde brasileiro, como a Política Nacional de Alimentação e Nutrição (PNAN)^1^, que estabelece a organização do cuidado nutricional na RAS, e a Política Nacional de Atenção Básica^4^, que define a nutrição como ação transversal da Estratégia Saúde da Família (ESF), o modelo ideal da APS abrangente ainda enfrenta barreiras. Os inúmeros programas e ações de AN caracterizam-se pela permanência de uma lógica assistencial programática.

As ações programáticas são caracterizadas pela organização do serviço em torno de agravos específicos e normas técnicas prescritivas, impondo barreiras à implantação de um modelo assistencial pautado na integralidade. Isso ocorre devido à tendência dessas ações em fragmentar o cuidado e à sua baixa capacidade de adaptação aos diferentes contextos político-organizacionais, socioeconômicos e demográficos dos territórios^6^.

A persistência da lógica programática é particularmente crítica diante do cenário de transição nutricional brasileiro, marcado pela coexistência da desnutrição e do crescimento acelerado da obesidade e outras doenças crônicas não transmissíveis. A ausência de uma atenção nutricional efetivamente implantada na APS resulta em uma assistência reativa e centrada na doença, incapaz de responder aos problemas de saúde contemporâneos. Assim, é fundamental desenvolver modelos avaliativos da implantação da atenção nutricional, enquanto parte do processo de trabalho das equipes e de responsabilidade da gestão municipal, com base nos preceitos da APS abrangente e passível de aplicação em diferentes contextos.

A relevância de avaliar a implantação no âmbito municipal reside no fato de ser este o nível de gestão responsável pela execução direta das ações e pela organização da rede de cuidado. Compreender como diferentes municípios, com capacidades instaladas e contextos socioeconômicos heterogêneos, interpretam e operacionalizam as diretrizes nacionais de AN é fundamental para identificar gargalos institucionais. Definir o grau de implantação (GI) torna-se uma etapa prioritária, uma vez que não se pode assegurar a eficácia das intervenções sem antes determinar como as características organizacionais locais favorecem ou limitam a dinâmica da assistência nutricional^9^.

Nesta perspectiva, o objetivo deste estudo foi avaliar a implantação da atenção nutricional no âmbito da APS em municípios brasileiros e identificar a associação do GI com aspectos do contexto sociodemográfico e político-organizacional.

## MÉTODOS

Trata-se de uma pesquisa avaliativa com abordagem quantitativa, do tipo análise de implantação, que tem como objetivo determinar o GI em diferentes contextos e identificar a influência do contexto sobre o GI^10,11^.

O modelo avaliativo foi definido em consenso com grupos de especialistas em 2017, orientado pela responsabilidade dos gestores municipais em garantir as condições estruturais, organizacionais e o provimento da atenção nutricional na perspectiva de uma APS abrangente. O modelo avaliativo com o detalhamento sobre o consenso encontra-se publicado em Machado et al.^12^ A matriz de análise de julgamento, composta por duas dimensões, seis subdimensões, 17 indicadores, 33 medidas e seus respectivos parâmetros, está apresentada no Quadro.

**Quadro:** Matriz de análise e julgamento da atenção nutricional na APS com os parâmetros utilizados nas medidas e indicadores.

Subdimensão	Indicadores	Medidas	Parâmetros	Agregação^a^	Fonte
**DIMENSÃO 1 – GESTÃO**					
1.1 Infraestrutura	1.1.1 Adequação de espaço físico na UBS para a atenção em saúde	Percentual de UBS com sala de atividades coletivas	3º quartil: bom Interv: regular 1º quartil: ruim^b^	Ambos bom (10) Demais regular (5) Ambos ruim (0)	PMAQ
		Percentual de UBS com disponibilidade de consultório equipado com computador e acesso à internet para atendimento clínico	3º quartil: bom Interv: regular 1º quartil: ruim^b^		PMAQ
	1.1.2 Disponibilidade de equipamentos para as ações de AN	Percentual de UBS com disponibilidade de equipamentos em condições de uso para as ações de alimentação e nutrição (balança antropométrica adulto 150/200kg, balança infantil, fita métrica, régua antropométrica)	100%: bom < 100%: ruim	Bom (10) Ruim (0)	PMAQ
	1.1.3 Disponibilidade de insumos para assistência nutricional	Percentual de UBS com disponibilidade de suplementos alimentares (sais para reidratação oral, sulfato ferroso, ácido fólico)	100%: bom < 100%: ruim	Bom (10) Ruim (0)	PMAQ
1.2 Recursos Humanos	1.2.1 Suficiência de pessoal na APS	Cobertura populacional estimada das equipes de atenção básica (ESF e parametrizadas)^c^	Até 10 mil hab. = 100%: bom Entre 10 e 50 mil hab. = 80%: bom Acima de 50 mil hab. = 60%: bom	Ambos bom (10) Demais regular (5) Ambos ruim (0)	CNES/IBGE
		Percentual de equipes mínimas completas no município	100%: bom < 100%: ruim		CNES/PMAQ
	1.2.2 Suporte de outras categorias profissionais para as ações de AN	Percentual de UBS que contam com profissionais de saúde para além da equipe mínima (apoio do nutricionista e pelo menos mais um profissional entre assistente social ou psicólogo ou educador físico)	3º quartil: bom Interv: regular 1º quartil: ruim^b^	Ambos bom (10) Demais regular (5) Ambos ruim (0)	PMAQ
		Percentual de equipes que possuem apoio matricial (eMulti ou CAPS ou especialistas da rede ou vigilância em saúde ou polos de academia da saúde)	3º quartil: bom Interv: regular 1º quartil: ruim^b^		PMAQ
1.3 Organização e Normatização	1.3.1 Condições para o acesso à saúde	Percentual de UBS com disponibilidade de atendimento no horário de almoço ou estendido no período noturno	50%: bom < 50%: ruim	3 “bom” = bom (10) 2 “bom” = regular (5) ≤ 1 “bom” = ruim (0)	PMAQ
		Percentual de equipes que realiza agendamento de consultas em qualquer dia e horário de forma presencial ou por telefone	100%: bom < 100%: ruim		PMAQ
		Percentual de equipes que realiza avaliação de risco e vulnerabilidade dos usuários no acolhimento	≥ 90%: bom 89% a 75%: regular < 75%: ruim		PMAQ
	1.3.2 Fluxos de encaminhamento para os diferentes pontos de atenção	Percentual de equipes que recebem auxílio de outros profissionais da rede para a resolução de casos considerados complexos	≥ 90%: bom 89% a 75%: regular < 75%: ruim	Ambos bom (10) Demais regular (5) Ambos ruim (0)	PMAQ
		Percentual de equipes que realizam encaminhamento para usuários com obesidade para serviço especializado	≥ 90%: bom 89% a 75%: regular < 75%: ruim		
		Percentual de equipes que obtêm retorno da avaliação realizadas pelos especialistas da rede	≥ 90%: bom 89% a 75%: regular < 75%: ruim		PMAQ
	1.3.3. Padronização da atenção à saúde	Percentual de equipes que utiliza protocolos de estratificação de risco para (hipertensão arterial sistêmica, diabetes mellitus, crianças menores de 2 anos)	≥ 90%: bom 89% a 75%: regular < 75%: ruim	Bom (10) Regular (5) Ruim (0)	PMAQ
	1.3.4 Disponibilidade de informação para a atenção nutricional	Atualização dos sistemas de informação para consumo alimentar de todos os ciclos de vida (Sisvan *Web*) com regularidade no último quadriênio (2011–2014)	Sim: bom Não: ruim	3 “bom” = bom (10) 2 “bom” = regular (5) 1 ou zero “bom” = ruim (0)	Sisvan *Web*
		Atualização dos sistemas de informação para estado nutricional de todos os ciclos de vida (Sisvan *Web*) com regularidade no último quadriênio (2010–2014)	Sim: bom Não: ruim		Sisvan *Web*
		Percentual de crianças menores de cinco anos com registro de estado nutricional no Sisvan *Web*^d^	≥ 80%: bom < 80%: ruim		Sisvan *Web*/IBGE
**DIMENSÃO 2 – ASSISTÊNCIA**					
2.1 Vigilância Alimentar e Nutricional	2.1.1 Acompanhamento contínuo do estado nutricional e consumo alimentar	Percentual de equipes que realizam ações de vigilância alimentar e nutricional	100%: bom < 100%: ruim	3 “bom” = bom (10) 2 “bom” = regular (5) ≤ 1 “bom” = ruim (0)	PMAQ
		Percentual de equipes que monitoram índices de aleitamento materno e alimentação complementar saudável	100%: bom < 100%: ruim		PMAQ
		Percentual de equipes que realizam avaliação nutricional na escola	3º quartil: bom Interv: regular 1º quartil: ruim^b^		PMAQ
	2.1.2 Planejamento local em saúde	Percentual de equipes que utilizam mapas com desenho do território	100%: bom < 100%: ruim	Ambos bom (10) Demais regular (5) Ambos ruim (0)	PMAQ
		Percentual de equipes que realiza o monitoramento e análise de indicadores e informações de saúde	≥ 90%: bom 89% a 75%: regular < 75%: ruim		PMAQ
2.2 Promoção da Alimentação Adequada e Saudável	2.2.1 Desenvolvimento de atividades de educação alimentar e nutricional	Percentual de equipes que realizam ações de educação alimentar e nutricional sobre aleitamento materno e alimentação saudável	100%: bom < 100%: ruim	Ambos bom (10) Demais regular (5) Ambos ruim (0)	PMAQ
		Percentual de equipes que convida os usuários com obesidade para participar de grupos de promoção à saúde sobre alimentação saudável e atividade física	100%: bom < 100%: ruim		PMAQ
	2.2.2 Articulação intersetorial no território	Percentual de equipes que realizam atividades de promoção à saúde na escola	3º quartil: bom Interv: regular 1º quartil: ruim^b^	Ambos bom (10) Demais regular (5) Ambos ruim (0)	PMAQ
		Percentual de equipes que desenvolvem ações de segurança alimentar e nutricional e promoção da alimentação adequada na escola	3º quartil: bom Interv: regular 1º quartil: ruim^b^		PMAQ
2.3 Controle e Acompanhamento de Agravos Nutricionais	2.3.1 Solicitação de exames de diagnóstico na APS	Percentual de equipes que solicita exames clínicos na rede de serviços (creatinina, perfil lipídico, hemoglobina glicosilada, glicemia de jejum no pré-natal, dosagem de hemoglobina e hematócrito no pré-natal)	≥ 90%: bom 89% a 75%: regular < 75%: ruim	Bom (10) Regular (5) Ruim (0)	PMAQ
	2.3.2 Organização da atenção segundo o risco nutricional	Percentual de equipes que organizam a agenda de acordo com o risco classificado para usuários (gestantes, hipertensos, diabéticos, obesidade, crianças até 2 anos)	≥ 90%: bom 89% a 75%: regular < 75%: ruim	3 “bom” = bom (10) 2 “bom” = regular (5) ≤ 1 “bom” = ruim (0)	PMAQ
		Percentual de equipes que organizam consultas de acompanhamento para usuários diagnosticados com obesidade	≥ 90%: bom 89% a 75%: regular < 75%: ruim		PMAQ
		Percentual de equipes que realizam busca ativa de crianças em desenvolvimento e com baixo peso e possuem registro das informações de desenvolvimento e estado nutricional	100%: bom < 100%: ruim		PMAQ
	2.3.3 Atenção interdisciplinar e multiprofissional	Percentual de equipes que aciona o apoio de outros profissionais para o acompanhamento dos usuários com obesidade	3º quartil: bom Interv: regular 1º quartil: ruim^b^	Ambos bom (10) Demais regular (5) Ambos ruim (0)	PMAQ
		Percentual de equipes que realizam ações compartilhadas com organização da agenda de forma conjunta entre todos os profissionais que integram a equipe	≥ 90%: bom 89% a 75%: regular < 75%: ruim		PMAQ
	2.3.4 Coordenação do cuidado	Percentual de equipes que possuem registro de usuários com hipertensão, diabetes, obesidade e gestantes em maior risco encaminhados a outros pontos de atenção	≥ 90%: bom 89% a 75%: regular < 75%: ruim	Ambos bom (10) Demais regular (5) Ambos ruim (0)	PMAQ
		Percentual de equipes que realiza gestão de casos que necessitam de outros pontos de atenção	≥ 90%: bom 89% a 75%: regular < 75%: ruim		PMAQ

Interv.: intervalo entre 1º e 3º quartil; AN: alimentação e nutrição; APS: Atenção Primária à Saúde; ESF: Estratégia Saúde da Família; UBS: unidade básica de saúde; PMAQ: Programa de Melhoria do Acesso e da Qualidade da Atenção Básica; eMulti: Equipes Multiprofissionais na Atenção Primária à Saúde; CAPS: Centro de Atenção Psicossocial; IBGE: Instituto Brasileiro de Geografia e Estatística; CNES: Cadastro Nacional de Estabelecimentos de Saúde; Sisvan: Sistema Nacional de Vigilância Alimentar e Nutricional.

^a^ As medidas foram agregadas para compor a pontuação de cada indicador.

^b^ Parâmetros definidos segundo a distribuição quartil para cada estrato de porte populacional.

^c^ Parâmetro definido conforme Portaria nº 1.007/2010 para classificação de incentivos financeiros e Portaria Nº 2.355/2013 para média recomendada de 3.000 habitantes por equipe. Ambas publicadas pelo Ministério da Saúde.

^d^ Considerou-se a média nacional de cobertura vacinal de 83%.

Foram utilizados dados secundários de diferentes sistemas de informação nacionais, como o sítio eletrônico do Ministério da Saúde para acesso ao resultado do segundo ciclo da avaliação externa do Programa de Melhoria do Acesso e da Qualidade da Atenção Básica (PMAQ-AB) entre 2013–2014, do Cadastro Nacional de Estabelecimentos de Saúde (CNES) para verificação das unidades básicas de saúde (UBS), do Instituto Brasileiro de Geografia e Estatística para o porte populacional dos municípios (2010) e do Sistema Nacional de Vigilância Alimentar e Nutricional (Sisvan) (2011–2014) para verificar a atualização dos dados sobre o estado nutricional e o consumo alimentar. Para as variáveis de contexto, foram consultados adicionalmente os bancos de dados do Programa das Nações Unidas para o Desenvolvimento (PNUD Brasil) e a estratificação dos municípios brasileiros segundo as condições que influenciam a gestão em saúde disponibilizada pelo Núcleo de Extensão e Pesquisa em Avaliação em Saúde da Universidade Federal de Santa Catarina^13^.

Considerando o caráter voluntário da adesão ao PMAQ-AB e o potencial de seleção estratégica de unidades com melhor desempenho pelos gestores municipais, adotou-se como critério de elegibilidade a inclusão apenas de municípios com participação igual ou superior a 80% do total de suas UBS e equipes existentes no CNES. Tal decisão metodológica visou reduzir o viés de seleção e garantir maior consistência interna na representação da análise municipal, conforme utilizado em estudo avaliativo publicado anteriormente^14^. Em relação à definição pelo segundo ciclo do PMAQ, esta se justifica tendo em vista a completude de informações relacionadas à atenção nutricional, objeto da avaliação.

As medidas e os indicadores foram ajuizados segundo os parâmetros definidos na matriz de análise de julgamento (Quadro). Buscando garantir a comparabilidade entre grupos homogêneos de municípios, algumas medidas foram ajuizadas segundo distribuição quartil por porte municipal. O juízo de valor (JV) para as subdimensões, dimensões e GI foi determinado pela diferença percentual entre a pontuação máxima (PM) e a pontuação observada (PO), utilizando a fórmula JV = (∑ PO/ ∑ PM) X 100. Os indicadores, subdimensões e dimensões foram classificados como “bom” quando atingiram valores acima de 66,6% da PM, como “ruim” quando alcançaram até 33,3% e como “regular” nas demais situações. Foi atribuído peso 2 para a PO na dimensão *Assistência*, pela sua maior aproximação com o objeto avaliativo.

A classificação do GI da atenção nutricional na APS seguiu a metodologia proposta por Vieira da Silva et al.^15^, na qual se consideraram as categorias de GI segundo os pontos de corte da distribuição em tercis, adotando-se os critérios: (1) Incipiente: valores > 0 e < 33,3%; (2) Intermediário: valores > 33,3 e < 66,6%; (3) Avançado: valores > 66,6%.

A análise descritiva contemplou a distribuição dos municípios segundo o JV das dimensões, subdimensões e indicadores e também segundo as categorias do GI e as variáveis de contexto. Foi realizado teste qui-quadrado para verificar os fatores associados com a implantação da atenção nutricional e definir as variáveis elegíveis (p < 0,20) para o modelo de regressão.

As variáveis independentes utilizadas foram: Porte Municipal (> 100.000 habitantes/ entre 25.000 e 100.000/ < 25.000); Região (Norte/Nordeste/Sudeste/Sul/Centro-Oeste); Índice de Desenvolvimento Humano Municipal (IDHm) (tercil); produto interno bruto (PIB) *per capita* (tercil); Proporção da população menor que cinco anos (tercil) para o Bloco 1 - Aspectos socioeconômicos e demográficos. E as variáveis: Estratos municipais segundo a capacidade para gestão em saúde; Recebimento de recursos para as ações de AN (Não/Sim); Cadastro de gestor para as ações de AN (Não/Sim); Apoio do nutricionista na APS (Não/Sim) e Cobertura da ESF compuseram o Bloco 2 - Aspectos Político-Organizacionais.

Em relação aos estratos municipais segundo a capacidade para gestão em saúde, essa classificação é realizada a partir da associação das características demográficas (densidade demográfica e taxa de urbanização), capacidade de financiamento (PIB *per capita*) e poder aquisitivo da população (percentual de extrema pobreza e cobertura de plano de saúde), que somados definem as condições para a gestão do sistema local de saúde em três classes: favorável, regular ou desfavorável. As três classes associadas ao porte populacional dos municípios classifica-os em sete estratos^13^, sendo eles:

- Pequeno desfavorável: municípios com até 25.000 habitantes e influentes desfavoráveis para gestão;
- Pequeno regular: municípios com até 25.000 habitantes e influentes regulares para gestão;
- Pequeno favorável: municípios com até 25.000 habitantes e influentes favoráveis para gestão;
- Médio desfavorável: municípios com 25.000 a 100.000 habitantes e influentes desfavoráveis para gestão;
- Médio regular: municípios com 25.000 a 100.000 habitantes e influentes regulares para gestão;
- Médio favorável: municípios com 25.000 a 100.000 habitantes e influentes favoráveis para gestão;
- Grande: municípios com mais de 100.000 habitantes, independentemente dos influentes.

O desfecho foi dicotomizado em “*implantado* (1) e *não implantado* (0)*”* para os testes de associação com os dois blocos de variáveis independentes. Como medida de associação, foram calculadas as *odds ratio* (OR), com os seus respectivos intervalos de 95% de confiança (IC95%) por meio de regressão logística multivariada.

Foram criados dois modelos de regressão ajustados às variáveis do bloco 1 e do bloco 2, utilizando teste de probabilidade em cada bloco para identificação dos subgrupos de categorias com maior e menor chance de implantação da atenção nutricional nos municípios. O diagnóstico do modelo deu-se pelo teste de qualidade de ajuste “*goodness-of-fit”.* As categorias com menos de 1% de prevalência do desfecho foram removidas nas variáveis porte municipal e estratos municipais, segundo o desempenho para a gestão para os modelos de regressão.

Os dados foram processados em planilha no Microsoft Office Excel^®^ (Microsoft Corp., Estados Unidos) e, em seguida, transferidos para o *software* estatístico Stata^®^ 13.0 (*Stata Corp, College Station*, Estados Unidos). Em função das etapas metodológicas de consenso e envolvimento de *stakeholders*^12^, esta pesquisa foi aprovada pelo Comitê de Ética da Universidade Federal de Santa Catarina, em conformidade com a Resolução nº 466/2012 do Ministério da Saúde, sob número de parecer 2.047.153/2017.

## RESULTADOS

Foram avaliados 4.064 municípios brasileiros, correspondendo a 73% dos municípios brasileiros em 2014. A amostra apresenta distribuição homogênea, com percentual de representação acima de 50% na maioria dos estados, com exceção do Maranhão (15,7%), do Acre (31,8%) e de Roraima (40,0%).

A atenção nutricional na APS apresenta níveis distintos de implantação nos municípios analisados. A maioria (57,7%) encontra-se em estágio intermediário, enquanto 30,7% estão em nível incipiente e apenas 11,6% em nível avançado. Regionalmente, Santa Catarina (24,3%) e Rio Grande do Sul (23,5%) apresentam o maior percentual de municípios com implantação avançada. Em contraste, nove estados, incluindo Acre, Amazonas, Roraima, Rondônia, Pará, Alagoas, Mato Grosso do Sul, Espírito Santo e Rio de Janeiro, não registraram nenhum município com implantação da atenção nutricional na APS em nível avançado (Figura).

**Figura:** Distribuição percentual de municípios com atenção nutricional na Atenção Primária à Saúde implantada em nível avançado, em relação ao total de municípios avaliados, segundo as unidades da federação (n = 4.064).

BR: Brasil; AC: Acre; AM: Amazonas; RR: Roraima; RO: Rondônia; AP: Amapá; PA: Pará; TO: Tocantins; MA: Maranhão; PI: Piauí; CE: Ceará; RN: Rio Grande do Norte; PB: Paraíba; PE: Pernambuco; AL: Alagoas; SE: Sergipe; BA: Bahia; MT: Mato Grosso; GO: Goiás; MS: Mato Grosso do Sul; MG: Minas Gerais; ES: Espírito Santo; SP: São Paulo; RJ: Rio de Janeiro; PR: Paraná; SC: Santa Catarina; RS: Rio Grande do Sul.

Comparando as duas dimensões de análise da matriz avaliativa, a gestão e a assistência, pode-se verificar pior resultado na dimensão da *Gestão*. Essa dimensão analisa a garantia de condições estruturais e organizacionais para o provimento das ações de AN e apresentou apenas 8,2% dos municípios classificados como “bom”. Analisando as subdimensões, aspectos de *Infraestrutura* e *Recursos Humanos* foram considerados satisfatórios em pouco mais de um terço dos municípios, 36,2% e 33,7%, respectivamente. Menos de 1% alcançou os parâmetros necessários para a classificação “bom” na subdimensão *Organização e Normatização* (Tabela 1).

**Tabela 1:** Distribuição de municípios avaliados para implantação da atenção nutricional na APS segundo os indicadores, subdimensões e dimensões com juízo de valor utilizados (n = 4.064).

Dimensão	Classificação					
Subdimensão	Ruim		Regular		Bom	
Indicadores	n	%	n	%	n	%
**Gestão**	**1.280**	**31,5**	**2.451**	**60,3**	**333**	**8,2**
Infraestrutura	879	21,6	1.715	42,2	1.470	36,2
Adequação de espaço físico	759	18,7	2.628	64,7	677	16,7
Disponibilidade de equipamentos	1.135	27,9	-	-	2.929	72,1
Disponibilidade de insumos	2.674	65,8	-	-	1.390	34,2
Recursos Humanos	1.016	25,0	1.678	41,3	1.370	33,7
Suficiência de pessoal	779	19,2	2.072	51,0	1.213	29,8
Suporte de outras categorias profissionais	698	17,2	2.692	66,2	674	16,6
Organização e normatização	2.741	67,5	1.288	31,6	35	0,9
Condições para o acesso à saúde	2.639	64,9	1.311	32,3	114	2,8
Fluxos de encaminhamento	1.505	37,0	2.128	52,4	431	10,6
Padronização da atenção à saúde	2.477	61,0	316	7,7	1.271	31,3
Disponibilidade de informação	3.669	90,2	368	9,1	27	0,7
**Assistência**	**1.370**	**33,7**	**1.894**	**46,6**	**800**	**19,7**
Vigilância Alimentar e Nutricional	975	24,0	979	24,1	2.110	51,9
Acompanhamento contínuo	1.273	31,3	1.465	36,1	1.326	32,6
Planejamento local em saúde	334	8,2	1.511	37,2	2.219	54,6
Promoção da Alimentação Adequada e Saudável	2.038	50,2	908	22,3	1.118	27,5
Desenvolvimento de atividades de EAN	2.289	56,3	1.156	28,4	619	15,2
Articulação intersetorial no território	763	18,8	1.923	47,3	1.378	33,9
Controle e Acompanhamento dos Agravos Nutricionais	1.473	36,3	2.163	53,2	428	10,5
Solicitação de exames de diagnóstico na APS	1.094	26,9	499	12,3	2.471	60,8
Organização da atenção segundo o risco nutricional	3.205	78,9	614	15,1	245	6,0
Atenção interdisciplinar e multiprofissional	730	18,0	2.687	66,1	647	15,9
Coordenação do cuidado	2.132	52,5	1.580	38,9	352	8,7

EAN: educação alimentar e nutricional; APS: Atenção Primária à Saúde.

Analisando cada subdimensão separadamente, na *Infraestrutura* destaca-se positivamente o indicador “Disponibilidade de equipamentos para as ações de AN”, com a maioria dos municípios apresentando o melhor resultado (72,1%). Em contrapartida, a “Adequação de espaço físico” foi classificada como “regular” na maioria dos municípios (64,7%), enquanto a “Disponibilidade de insumos” apresentou o cenário mais crítico, sendo considerada “ruim” em 65,8% da amostra.

Na subdimensão *Recursos Humanos,* mais da metade dos municípios apresentou resultado regular para os indicadores “Suficiência de Pessoal” (51%) e “Suporte de outras categorias profissionais para as ações de AN” (66,2%).

Entre os indicadores que compõem a subdimensão *Organização e Normatização* encontram-se os piores resultados para os aspectos da gestão municipal. Mais de 60% dos municípios são classificados como “ruim” para as “Condições para o acesso à saúde” (64,9%) e “Padronização da Atenção” (61%). Além desses, 90,2% dos municípios avaliados apresentaram desempenho “ruim” no indicador “Disponibilidade de Informação para a Atenção Nutricional”.

A dimensão *Assistência*, por sua vez, apresentou 19,7% dos municípios com bom desempenho. Nessa dimensão, são avaliados os aspectos sobre a organização da atenção nutricional realizada pelas equipes de saúde na APS. Comparando entre as subdimensões do bloco da Assistência, o destaque positivo foi para a *Vigilância Alimentar e Nutricional* com 51,9% dos municípios classificados como “bom”. Em contraponto, 50,2% dos municípios foram classificados como “ruim” na subdimensão *Promoção da Alimentação Adequada e Saudável* e, em *Controle e Acompanhamento dos Agravos Nutricionais*, poucos municípios foram avaliados como “bom” (10,5%).

Trazendo o foco para a subdimensão *Vigilância Alimentar e Nutricional* o destaque positivo foi para o indicador “Planejamento Local em Saúde”, com 54,6% dos municípios classificados como “bom”. Para o “Acompanhamento contínuo do estado nutricional e consumo alimentar” houve distribuição homogênea dos municípios entre os três critérios de avaliação.

Na *Promoção da Alimentação Adequada e Saudável,* a pior avaliação foi no indicador “Desenvolvimento de atividades de Educação Alimentar e Nutricional” com 56,3% dos municípios avaliados como “ruim” nesse aspecto. O segundo indicador que abordou a “Articulação Intersetorial do território” foi levemente superior, apresentando 47,3% dos municípios avaliados como “regular” e 18,8% como “ruim.”

Apesar do resultado insatisfatório para os aspectos que envolvem a subdimensão *Controle e Acompanhamento dos Agravos Nutricionais,* quando se avaliam separadamente os indicadores, destaca-se a “Solicitação de exames de diagnóstico na APS”, indicador com maior percentual de municípios considerados “bons” (60,8%). Em contraponto, situam-se os indicadores “Organização da atenção segundo o risco nutricional” e “Coordenação do Cuidado”, os quais apresentaram os menores percentuais classificados como “bom” na dimensão gestão, 6,0% e 8,7%, respectivamente (Tabela 1).

Na análise de contexto, com exceção dos fatores *Recebimento de recursos para as ações de* AN (2011–2013) e *Cadastro de gestor para as ações de* AN, as demais variáveis apresentaram associação estatisticamente significativa com o GI da atenção nutricional (p < 0,001) (Tabela 2). Os municípios com menos de 25.000 habitantes apresentaram 14,7% com GI avançado da atenção nutricional, e nenhum município acima de 100.000 habitantes foi classificado neste mesmo nível de implantação.

**Tabela 2:** Distribuição de municípios segundo grau de implantação e as características de contexto (n = 4.064).

Variável	Total	Grau de implantação da atenção nutricional			
		Incipiente	Intermediário	Avançado	p-valor^b^
**Contexto**	**n (%)**	**% (IC95%)**	**% (IC95%)**	**% (IC95%)**
Brasil	4.064 (100)	30,7	57,7	11,6	
*Bloco 1 - Aspectos socioeconômicos e demográficos*					
Porte Municipal					< 0,001
> 100.000	182 (4,5)	53,8 (46,5–61,1)	46,2 (38,8–53,5)	-	
Entre 25.000 e 100.000	742 (18,3)	51,6 (48,0–50,5)	46,9 (43,3–50,5)	1,5 (0,6–2,3)	
< 25.000 habitantes	3.140 (77,2)	24,4 (22,9–25,9)	60,9 (59,2–62,6)	14,7 (13,4–15,9)	
Região					< 0,001
Norte	269 (6,6)	56,5 (50,5–62,5)	38,3 (32,4–44,1)	5,2 (2,5–7,9)	
Nordeste	1.262 (31,1)	35,1 (32,5–37,7)	59,4 (56,6–62,1)	5,5 (4,3–6,8)	
Sudeste	1.239 (30,5)	23,7 (21,4–26,1)	62,6 (59,8–65,2)	13,7 (11,8–15,6)	
Sul	899 (22,1)	21,8 (19,1–24,5)	57,6 (54,4–60,5)	20,6 (17,9–23,2)	
Centro-Oeste	395 (9,7)	41,3 (36,4–46,1)	50,6 (45,7–55,6)	8,1 (5,4–10,8)	
IDHm^c^					< 0,001
0,418 a 0,625	1.358 (33,4)	35,8 (33,2–38,3)	57,6 (55,0–60,3)	6,6 (5,2–7,9)	
0,626 a 0,704	1.373 (33,8)	29,6 (27,2–32,0)	57,3 (54,7–60,0)	13,1 (11,3–14,9)	
0,705 a 0,862	1,333 (32,8)	26,7 (24,3–29,1)	58,1 (55,5–60,8)	15,2 (13,2–17,1)	
PIB *per capita*^c^					< 0,001
≥ R$ 2.782,87	1.355 (33,3)	33,8 (31,2–36,2)	58,9 (56,3–61,5)	7,3 (5,9–8,8)	
≥ R$ 6.462,94	1.355 (33,4)	30,6 (28,2–33,1)	57,7 (55,1–60,3)	11,7 (10,0–13,4)	
≥ R$ 13.436,55	1.354 (33,3)	27,8 (25,4–30,2)	56,5 (53,9–59,1)	15,7 (13,8–17,7)	
Proporção de população < 5 anos^c^					< 0,001
≥ 2,3%	1.355 (33,3)	20,9 (18,7–23,1)	60,0 (57,4–62,6)	19,1 (17,0–21,2)	
≥ 6,5%	1.355 (33,4)	31,0 (28,5–33,5)	59,1 (56,5–61,7)	9,9 (8,2–11,5)	
≥ 7,7%	1.354 (33,3)	40,3 (37,6–42,9)	54,0 (51,3–56,6)	5,7 (4,5–7,0)	
*Bloco 2 - Aspectos político-organizacionais*					
Estratos municipais segundo a capacidade para gestão em saúde^a^					< 0,001
Grande	172 (4,2)	52,9 (45,4–60,4)	47,1 (39,5–54,6)	-	
Médio favorável	268 (6,6)	41,8 (35,8–47,7)	56,0 (50,0–61,9)	2,2 (0,1–4,0)	
Médio regular	245 (6,0)	55,6 (49,2–61,8)	42,8 (36,6–49,1)	1,6 (0,1–3,2)	
Médio desfavorável	176 (4,3)	61,9 (54,7–69,2)	38,1 (30,8–45,3)	-	
Pequeno favorável	466 (11,5)	23,2 (19,3–27,0)	60,3 (55,8–64,7)	16,5 (13,1–19,9)	
Pequeno regular	1.495 (36,8)	22,7 (20,6–24,9)	60,6 (58,1–63,1)	16,7 (14,8–18,5)	
Pequeno desfavorável	1.241 (30,6)	28,3 (25,8–30,8)	60,8 (58,1–63,5)	10,9 (9,1–12,6)	
Recebimento de recursos para as ações de AN (2011–2013)					0,072
Não	88 (2,2)	22,7 (13,8–31,6)	59,1 (48,6–69,6)	18,2 (10,0–26,4)	
Sim	3.976 (97,8)	30,9 (29,4–32,3)	57,7 (56,1–59,2)	11,4 (10,4–12,4)	
Cadastro de gestor para as ações de AN					0,150
Não	2.691 (66,2)	31,4 (29,6–33,2)	56,7 (54,7–58,5)	11,9 (10,7–13,2)	
Sim	1.373 (33,8)	29,4 (26,9–31,8)	59,8 (57,2–62,4)	10,8 (9,2–12,5)	
Apoio de nutricionista na APS				< 0,001	
Não	2.442 (60,1)	36,8 (34,9–38,8)	55,0 (53,1–57,0)	8,2 (7,0–9,2)	
Sim	1.622 (39,9)	21,5 (19,5–23,5)	61,7 (59,4–64,1)	16,8 (15,0–18,7)	
Cobertura da ESF					< 0,001
Até 75,0%	565 (13,9)	46,3 (42,1–50,3)	47,4 (43,3–51,6)	6,3 (4,3–8,4)	
Entre 75,0% e 99,9%	484 (11,9)	45,7 (41,2–50,1)	49,8 (45,3–54,3)	4,5 (2,7–6,4)	
100%	3.015 (74,2)	25,4 (23,8–26,9)	60,9 (59,2–62,6)	13,7 (12,5–14,9)	

IDHm: Índice de Desenvolvimento Humano Municipal; PIB: produto interno bruto; AN: alimentação e nutrição; APS: Atenção Primária à Saúde; ESF: Estratégia Saúde da Família; IC95%: intervalo de confiança de 95%.

^a^ Proposta de estratificação municipal segundo a capacidade para a gestão em saúde segundo Calvo et al.^13^

^b^ Teste qui-quadrado.

^c^ Categorias referentes ao tercil de distribuição da amostra.

As regiões Sul e Sudeste apresentam os maiores percentuais de municípios com atenção nutricional implantada em estágio avançado, registrando 20,6% e 13,7%, respectivamente. Por outro lado, as regiões Norte e Centro-Oeste detêm as maiores proporções de implantação incipiente, com índices de 56,5% e 41,3%, respectivamente. Já no Nordeste, o grau intermediário predomina em 59,4% dos municípios avaliados.

Quando analisados o GI da atenção nutricional e sua relação com os valores de IDHm e PIB *per capita*, observa-se a relação diretamente proporcional com o desfecho, com maiores prevalências de implantação avançada à medida que aumentam o IDHm e o PIB *per capita*. Já a proporção de população menor de cinco anos foi inversamente proporcional ao GI, quanto menor a proporção de crianças no município maior é o GI da atenção nutricional (19,1%).

Entre os estratos municipais, segundo a capacidade de gestão, municípios classificados como “pequeno favorável” e “pequeno regular” são os que apresentaram maior prevalência para o GI avançado da atenção nutricional, aproximadamente 16,5%. Municípios que contam com o apoio do nutricionista na APS e 100% cobertura da ESF possuem mais que o dobro da implantação da atenção nutricional (Tabela 2).

A Tabela 3 apresenta os modelos de análise bruta e multivariada de regressão. Os aspectos socioeconômicos e demográficos apresentaram forte associação com a implantação da atenção nutricional na análise bruta. Municípios com menos de 25.000 habitantes e menor proporção de crianças de até cinco anos de idade apresentaram maiores prevalências de implantação da atenção nutricional, após o ajuste entre as variáveis. Apesar de as variáveis região e IDHm não permanecerem associadas, após o ajuste, as categorias região Sul e IDH maior que 0,705 mantiveram a associação.

**Tabela 3:** Associação entre as características de contexto e a implantação da atenção nutricional na APS (n = 4.064).

Variável	Atenção nutricional implantada			
	Bruta		Modelo	
Contexto	OR (IC95%)	p-valor	OR (IC95%)	p-valor
*Bloco 1 - Aspectos socioeconômicos e demográficos*^a^				
Porte Municipal		< 0,001		< 0,001
≥ 25.000 habitantes	1,00		1,00	
< 25.000 habitantes	14,25 (7,80–26,03)		14,11 (7,65–26,02)	
Região		< 0,001		0,996
Norte	1,00		1,00	
Nordeste	1,07 (0,59–1,93)		0,76 (0,39–1,47)	
Sudeste	2,90 (1,65–5,08)		1,55 (0,82–2,92)	
Sul	4,72 (2,69–8,28)		1,98 (1,03–3,81)	
Centro-Oeste	1,61 (0,84–3,07)		0,87 (0,43–1,74)	
IDHm^d^		< 0,001		0,143
0,418 a 0,625	1,00		1,00	
0,626 a 0,704	2,15 (1,65–2,81)		1,26 (0,81–1,94)	
0,705 a 0,862	2,55 (1,96–3,31)		1,30 (0,79–2,13)	
PIB *per capita*^d^		< 0,001		0,024
≥ R$ 2.782,87	1,00		1,00	
≥ R$ 6.462,94	1,66 (1,27– 2,15)		0,69 (0,45–1,04)	
≥ R$ 13.436,55	2,34 (1,82–30,1)		0,94 (0,60–1,47)	
Proporção de população < 5 anos^d^		< 0,001		< 0,001
≥ 2,3%	1,00		1,00	
≥ 6,5%	0,46 (0,37–0,58)		0,65 (0,51–0,84)	
≥ 7,7%	0,26 (0,20–0,34)		0,53 (0,38–0,76)	
*Bloco 2 - Aspectos Político-Organizacionais*^b^				
Estratos municipais segundo a capacidade para gestão em saúde^c^		< 0,001		< 0,001
Médio favorável	1,00		1,00	
Médio regular	1,20 (0,34–1,30)		1,08 (0,30–3,92)	
Pequeno favorável	9,86 (4,26–22,84)		8,77 (3,71–20,73)	
Pequeno regular	14,46 (6,38–32,72)		13,07 (5,62–30,40)	
Pequeno desfavorável	8,83 (3,87–20,15)		7, 91 (3,36–18,63)	
Recebimento de recursos para as ações de AN (2011–2013)		0,068		
Não	1,00			
Sim	0,58 (0,34–1,01)			
Cadastro de gestor para as ações de AN		0,292		
Não	1,00			
Sim	0,90 (0,73–1,10)			
Apoio de nutricionista na APS		< 0,001		< 0,001
Não	1,00		1,00	
Sim	2,29 (1,89–2,79)		2,40 (1,97–2,94)	
Cobertura da ESF		< 0,001		0,005
Até 75,0%	1,00		1,00	
Entre 75,0% e 99,9%	0,70 (0,41–1,21)		0,46 (0,28–0,87)	
100%	2,33 (1,64–3,32)		1,11 (0,76–1,64)	

IDHm: Índice de Desenvolvimento Humano Municipal; PIB: produto interno bruto; AN: alimentação e nutrição; APS: Atenção Primária à Saúde; ESF: Estratégia Saúde da Família; OR: *odds ratio*; IC95% intervalo de confiança de 95%.

^a^ Modelo 1 incluiu as variáveis com p < 0.05 na análise bruta, utilizando as variáveis do bloco 1 ajustadas entre si.

^b^ Modelo 2 incluiu as variáveis com p < 0.05 na análise bruta, utilizando as variáveis do bloco 2 ajustadas entre si.

^c^ Proposta de estratificação municipal segundo Calvo et al.^13^.

^d^ Categorias referentes ao tercil de distribuição da amostra.

Quando realizado teste de probabilidade entre as variáveis que permanecem estatisticamente significativas após o ajuste de cada bloco (p < 0,001), municípios com mais de 25.000 habitantes, com menor PIB *per capita* (1º tercil ≥ R$ 2.782,87) e maior proporção de menores de cinco anos (3º tercil ≥ 7,7%) têm menos de 0,1% de chance de implantação avançada da atenção nutricional. Municípios com menos de 25.000 habitantes, maior PIB *per capita* (3º tercil ≥ R$ 13.436,55) e menor proporção de menores de cinco anos (1º tercil ≥ 2,3%) têm 25,8% de chance de implantação da atenção nutricional (dados não apresentados em tabela).

Já os municípios sem apoio do nutricionista na APS, do estrato médio favorável, com até 75% de cobertura da ESF e que receberam recursos para as ações de AN, possuem 2,3% de chance de implantação avançada da atenção nutricional. Aqueles que contam com o apoio do nutricionista na APS, do estrato pequeno regular, com 100% de cobertura da ESF e que não receberam recursos para as ações de AN têm 36,0% de chance de implantação da atenção nutricional (dados não apresentados em tabela).

## DISCUSSÃO

A implantação da atenção nutricional na APS brasileira é predominantemente intermediária ou incipiente com as maiores fragilidades concentradas na dimensão da gestão municipal. O estudo revela que, enquanto a vigilância alimentar e nutricional (VAN) apresenta avanços, a organização estrutural e as ações de promoção da saúde ainda enfrentam barreiras críticas de execução. O sucesso da implantação mostrou-se fortemente condicionado a melhores indicadores socioeconômicos, ao pequeno porte populacional e à presença estratégica de nutricionista na APS e cobertura de ESF.

Na dimensão de Gestão, evidenciou-se baixa adequação dos municípios aos critérios elencados para *Infraestrutura*, *Recursos Humanos* e aspectos de *Organização e Normatização*, condições indispensáveis a outros eixos assistenciais da APS. Esse cenário reforça que a ampliação do acesso e a estruturação dos serviços de APS no país requerem o enfrentamento de desafios gerenciais crônicos para efetivar a mudança do modelo de atenção à saúde no Brasil^16^.

Entre esses desafios, na subdimensão de *Infraestrutura*, observa-se uma incoerência marcada pela alta disponibilidade de equipamentos em contraste com a escassez de insumos e a inadequação de espaços físicos. Esse cenário reflete o modelo de indução federal vigente no período (como o PMAQ-AB), que priorizou o repasse de recursos para aquisição de bens permanentes. No entanto, esse modelo encontrou barreiras na capacidade de planejamento logístico e no custeio municipal para a manutenção de materiais básicos^14^.

A maior parte dos municípios analisados não possuía sais para reidratação oral, sulfato ferroso e ácido fólico, insumos essenciais para assistência nutricional e que compõem a lista de medicamentos básicos da Relação Nacional de Medicamentos^17^. Quanto ao espaço físico, as medidas utilizadas apontavam para a necessidade de pelo menos um consultório equipado com computador e acesso à internet nas unidades de saúde. Essa infraestrutura se configurava como essencial para o preenchimento adequado das fichas de cadastro no Sisvan *Web,* além da consulta às bases de dados para utilização das informações no planejamento.

Esses achados evidenciam que a posse do recurso material, isoladamente, é insuficiente para consolidar a atenção nutricional na APS. A carência de espaço físico adequado nas UBS compromete o acolhimento e o cuidado nutricional, enquanto a falta de insumos contínuos limita a resolutividade das ações educativas e de vigilância, tornando os equipamentos subutilizados e as práticas assistenciais descontínuas no território^14^.

O resultado negativo para a subdimensão *Organização e Normatização*, principalmente na disponibilidade de informação para a atenção nutricional, é um achado crítico. Este indicador avaliou a atualização sistemática de dados sobre consumo alimentar e estado nutricional no Sisvan *Web*, além do monitoramento de peso e altura de crianças menores de cinco anos. Embora as normativas da PNAN e do Programa Bolsa Família estabelecessem esse acompanhamento como prioridade, os resultados da pesquisa sugerem que a coleta dos dados se restringia, frequentemente, para fins de repasse financeiro.

Esses achados podem ser justificados pela carência de protocolos estabelecidos e pela reduzida presença de nutricionistas na APS para realizar o diagnóstico nutricional^18^. Além disso, diferente da realidade atual, caracterizada pelo incentivo à interoperabilidade dos sistemas de informação, o cenário de 2014 era de fragmentação, com o Sisvan operando de forma isolada. Esses fatores contribuem para um registro administrativo, desvinculado da análise dos dados e do planejamento assistencial ou da intervenção precoce^19,20^, indicando fragilidade da gestão municipal em utilizar a VAN de forma estratégica.

Por fim, ainda nessa subdimensão, a baixa adequação nos itens de acesso à saúde e padronização da atenção revela barreiras que comprometem diretamente a resolutividade do serviço. A baixa oferta de horários estendidos e as dificuldades no agendamento evidenciam que o acesso organizacional ainda era marcado por rigidez estrutural. Simultaneamente, a incipiente utilização de protocolos de estratificação de risco para hipertensão, diabetes e saúde da criança indica que a atenção nutricional operava de forma reativa. Sem a padronização baseada na vulnerabilidade, a atenção nutricional perde sua capacidade preditiva e deixa de atuar como eixo estratégico nas RAS^1^.

Em relação à dimensão da Assistência, esta apresentou uma adequação discretamente superior, embora ainda restrita a uma parcela reduzida dos municípios. Nota-se, contudo, uma expressiva disparidade interna entre as subdimensões: enquanto a VAN desponta como o eixo mais consolidado no cotidiano da APS, as ações de Promoção da Alimentação Adequada e Saudável e o Controle e Acompanhamento dos Agravos Nutricionais revelam lacunas profundas. Esse desequilíbrio sugere que, no período analisado, a capacidade das equipes em monitorar a população não se traduzia em intervenções práticas de promoção da saúde ou no manejo clínico especializado, evidenciando uma assistência predominantemente diagnóstica.

Ao detalhar sobre a subdimensão da VAN, observa-se que seu desempenho superior é potencializado, em grande medida, pelos avanços no planejamento local. Esse resultado sugere que a lógica de territorialização e a cultura de monitoramento de indicadores de saúde, pilares da ESF e incentivadas por programas de indução financeira, estavam mais consolidadas nas rotinas das equipes^4,5^. No entanto, o monitoramento do aleitamento materno e as ações de saúde nas escolas, embora normatizados, enfrentam certa dificuldade de execução que podem estar atreladas à fragmentação das práticas assistenciais e à carência de suporte técnico especializado para converter o diagnóstico territorial em intervenção clínica direta^18^.

Por outro lado, as subdimensões voltadas à *Promoção da Alimentação Adequada e Saudável* e ao *Controle e Acompanhamento dos Agravos Nutricionais* revelam os maiores obstáculos para a resolutividade da assistência. A elevada inadequação no desenvolvimento de atividades de Educação Alimentar e Nutricional sugere que, no cenário de 2014, as práticas de promoção ainda enfrentavam dificuldades para superar modelos puramente prescritivos^21^. Importante salientar que as ações de promoção da alimentação adequada e saudável dirigidas ao público materno-infantil são fundamentais para o cuidado integral à saúde e possuem evidências para o potencial de reversão dos baixos índices de aleitamento materno^22,23^.

Essa lacuna torna-se ainda mais evidente no enfrentamento dos agravos nutricionais. Enquanto a capacidade de solicitar exames diagnósticos apresenta um desempenho satisfatório, a organização da atenção com utilização de protocolos de classificação de risco ou a responsabilização da APS pelos casos encaminhados a outros pontos de atenção está entre os pontos mais críticos da dimensão Assistência. Esse cenário indica a persistência de uma lógica biomédica centrada na identificação da doença, mas que falha gravemente em gerenciar o itinerário terapêutico do usuário de forma ordenada.

Alguns estudos corroboram esses achados, denotando o desafio da APS em estabelecer linhas de cuidado e a organização dos pontos de atenção de forma articulada e adequada às necessidades de saúde dos usuários^24,25^. Tal desarticulação reafirma que a vigilância, isolada da gestão clínica e de protocolos de estratificação, é insuficiente para produzir mudança real no perfil epidemiológico da população. Essa tendência ao modelo assistencial fragmentado sugere a persistência de uma APS seletiva^5^, na qual o acesso a exames não garante, por si só, a integralidade do cuidado nutricional.

Em relação às características de contexto, os municípios com menos de 25.000 habitantes, localizados na região Sul, com melhores indicadores sociais (IDH e PIB *per capita*) e menor proporção de crianças foram identificados com condições favoráveis à implantação da atenção nutricional na APS. Melhores resultados na avaliação em municípios de menor porte também foram encontrados em estudos de avaliação da saúde bucal^26^, da gestão para o planejamento em saúde^27^ e do processo de trabalho na APS^28^.

As disparidades regionais na implantação da atenção nutricional refletem desigualdades estruturais históricas do sistema de saúde brasileiro. A predominância de estágios avançados nas regiões Sul e Sudeste converge com o maior desenvolvimento socioeconômico e com a concentração de infraestrutura e força de trabalho especializada, especialmente de nutricionistas^29,30^. Em contraste, os elevados índices de implantação incipiente no Norte e Centro-Oeste evidenciam desafios logísticos – como as grandes distâncias territoriais e a precária conectividade para alimentação dos sistemas –, além da dificuldade de retenção de profissionais em áreas remotas. Tais fatores limitam a resolutividade das ações para além dos registros básicos de VAN, aprofundando os vazios assistenciais nessas regiões^29,31^.

Sobre o PIB e o IDHm, estudos evidenciaram que piores indicadores sociais estão associados com maiores taxas de mortalidade infantil e Internações por Causas Sensíveis à Atenção Primária^32^, e podem ter relação com a implantação incipiente da atenção nutricional na APS. O estudo dessas complexas mediações permite identificar onde e como devem ser feitas as intervenções, com o objetivo de reduzir as iniquidades em saúde e os pontos em que tais intervenções podem provocar maior impacto^33^.

A presença do nutricionista como profissional de apoio à APS e os municípios com 100% da população com cobertura da ESF também se mostraram fortemente associados à melhor implantação da atenção nutricional. Considera-se o nutricionista enquanto profissional de referência ao apoio matricial das ações de AN na APS^34^ e como fundamental para incorporação das ações de promoção da saúde em todas as fases da vida, qualificando as ações sobre alimentação saudável e segurança alimentar e nutricional^35^.

Apesar do recebimento de recursos específicos para a implantação da atenção nutricional na APS e da existência de gestor das ações de AN nas secretarias municipais de saúde, estas condições não expressaram melhor GI. Em estudo sobre a APS no Brasil, destaca-se que os principais desafios para o processo de implantação são o financiamento insuficiente e a formação profissional não orientada pelo modelo de atenção integral^36^.

## CONCLUSÃO

O estudo evidencia que a implantação da atenção nutricional na APS brasileira em 2014 encontrava-se em um estágio incipiente, marcado por avanços significativos nos aspectos da VAN, mas ainda limitado por fragilidades críticas na gestão e na coordenação do cuidado. O panorama revelado aponta para um “paradoxo assistencial”: os municípios demonstram competência técnica para o planejamento territorial e para a execução de procedimentos diagnósticos, como a solicitação de exames, mas falham sistematicamente na entrega de ações finalísticas, como a educação alimentar e nutricional e o manejo clínico dos agravos nutricionais.

O baixo GI da atenção nutricional na APS, diante da complexidade dos contextos existentes no país, não minimiza as contribuições da APS para a saúde da população, nem o seu potencial na reorganização dos serviços de saúde e de um novo modelo assistencial. Por outro lado, indicam a necessidade de processos contínuos de avaliação e monitoramento das intervenções, qualificação da assistência e inserção da atenção nutricional como parte do modelo assistencial. A identificação de municípios com maior e menor chance de implantação da atenção nutricional possibilita o planejamento de políticas que promovam maior equidade na distribuição dos recursos em saúde.

Essa realidade sugere que a indução federal por meio de programas de melhoria da qualidade foi eficaz em instituir uma cultura de monitoramento e registro, porém insuficiente para romper com a lógica de uma APS seletiva, ainda centrada em modelos biomédicos e prescritivos. A acentuada desarticulação entre a coleta de dados e a prática clínica reafirma a necessidade urgente de fortalecer o papel do nutricionista no território, não apenas como um suporte técnico eventual, mas como articulador essencial na RAS com ações mais abrangentes e resolutivas.

Por fim, os resultados indicam que o fortalecimento da atenção nutricional no SUS depende da superação das ações fragmentadas, do isolamento dos sistemas de informação e da rigidez dos modelos organizacionais. Espera-se que a trajetória de integração tecnológica iniciada no período analisado evolua para uma gestão estratégica da informação, capaz de transformar dados epidemiológicos em direcionamentos para o cuidado integral. Superar as disparidades regionais e garantir que a VAN se traduza em promoção da saúde permanecem, portanto, como os principais desafios para a consolidação da PNAN no cenário contemporâneo.
